# Supplementary material for: Effect of mobile food environments on fast food visits
Source: Nat Commun. 2024 Mar 14;15:2291. doi: 10.1038/s41467-024-46425-2 (PMC10937966; doi:10.1038/s41467-024-46425-2)
Supplement: Supplementary file 1 — Supplementary Information [file 41467_2024_46425_MOESM1_ESM.pdf]

Supplementary Information for

Effect of mobile food environments on fast food visits

B. Garcia-Bulle, A. L. Horn, B. M. Bell, M. Bahrami, B. Bozkaya,
A. Pentland, K. de la Haye, E. Moro*

*Corresponding author: esteban.moroegido@gmail.com

Supplementary Notes

Supplementary Note 1: Data **6**
1.1 Extracting stays 6
1.2 Attribution of stays to POIs 6
1.3 Identifying home 6
1.4 Definition of metro areas 7
1.5 Descriptive analysis of the data 7
1.6 Other data used 9

Supplementary Note 2: Representativity **10**

Supplementary Note 3: Classification of Food Outlets **11**

Supplementary Note 4: Definition of context and food visit **13**

Supplementary Note 5: Detecting change of context **13**

Supplementary Note 6: Estimation of the effect of changing contexts **13**

Supplementary Note 7: Models **14**
7.1 Demographic models for exposure to home and mobile food environments and FFO visits 14
7.2 Model for visits to FO during Lunchtime 16
7.3 Model for visits to FO by socio-demographic traits 19
7.4 Model for the visits to FO after DMV 21
7.5 Model for the visits to FO during the whole day 21

Supplementary Note 8: Topic analysis of census tracts **22**

Supplementary Note 9: Effect of interventions on other health risk groups **25**

Supplementary Note 10: Main results by city **27**

Supplementary Note 11: Impact of mobile food environments for other types of food. **29**

Supplementary Note 12: Robustness checks 31
12.1 Different definitions of POI visit 31
12.2 Different definitions of the context 31
12.3 Different characterization of the context 32
12.4 Different sets of FO and FFO 33

Supplementary Note 13: Interventions 34

Supplementary Note 14: Libraries used 35

List of Supplementary figures

S1 A: Distribution of stay duration (in minutes) in our dataset for all POI, only those attributed to food POI and fast food POI. Vertical lines correspond to the minimum (5 min) and maximum (2 hours) used in our analysis. B: Density of the number of stays per user and type of POI. The vertical line corresponds to 50 stays used in our models for individuals. C: Density of the ratio of FFO to FO visited for each individual. D: Density of the mobile food environments and home environments for each individual. 8

S2 A) Correlation between the smartphone population detection in our mobility data and the official census population by census tract. B) Coefficients of the logistic regression coefficient without (raw) and with (weighted) weights in the regression proportional to $1/w_{\Omega_i}$. C: Effect of the different interventions re-weighted by $1/\Omega_i$ to account for the different ratio of users to the population in each census tract Ω. 10

S3 Comparison between the number of FFO by chain in different cities obtained from our Foursquare dataset and Infogroup's US Historical Business Data for 2016 (17). . . . 12

S4 Method to detect the change of context before lunch. We construct a binary time series for each of the census tracts for those users that have more than 30% of their contexts in two of them. We use a statistical test to detect statistically significant changes in the mean of those time series to know if and where changepoints happened. For the user in panel A, a significant change in the means (in red) is detected in December. However, for the user in panel B, no significant change is detected, and the user changes often between contexts. Statistically significant changes were detected using the `changepoint` library with a manual method and penalty of $n^{1/10}$ (see details of those methods in (18)). 14

S5 A) Correlogram between the different demographic variables used in our regressions. B) Result of the multivariate regression for the home and mobile food environments and the fraction of visits to FFO fitting a model using all demographic variables, see Eq. (2). C) Result of the univariate regression for the home and mobile food environments and the fraction of visits to FFO using only a given demographic variable (see text). 16

S6 A: Spatial autocorrelation of $\phi(x)$, as a function of the distance in km. B: change in odds of visit to an FFO at different distances between the context and the lunch action when the context includes 10% more FFO, obtained from the results in Table S6. C: Distribution of the distance between the context and the lunch action. The vertical dashed line corresponds to $d_{it} = \exp(3.335/2.091)$, see Table S6, and the horizontal line in B corresponds to the value of the model in the main text for all distances ($\sim$ 20%). 19

S7 Left: Effect of the mobile food context ϕ in choosing an FFO using logistic regression and hot-encoding different values of ϕ. Error bars are standard errors of the coefficients. Right: Effect of the mobile food context in choosing an FFO restricted to the food outings of different socio-demographic groups. Groups are selected as quantiles of each socio-demographic variable. 20

S8 Importance of the FFO context variable $\phi(\mathbf{c}_{it})$ for models built with actions at different hours of the data. Variable importance is given by the absolute value of the t-statistic of the variable in the regression model (2) by the hour. 21

S9 Correlation between the predictions for the total number of FFO visits (A and B) and fraction of FFO visits (C and D) of our model Eq. (2) and real data for the train (A and C) and test (B and D) sets in the two different out-of-sample strategies. 23
S10 Variation of different metrics to estimate the optimal number of topics in the different census tracts. Metrics were obtained using the `ldatuning` package (23). As we can see, several metrics show a minimum/maximum around $k = 20$. 24
S11 Composition of the different $k = 20$ POI topics found in our census tracts. We have given names to each of them for easy recognition. Bars represent the probability of finding each POI category in each of the topics. Note: no mobility data or visits were used in this analysis, only the spatial distribution of POIs from the Foursquare API. . 26
S12 Average (and standard error of the mean) frequency of each topic across all census tracts in our dataset. 27
S13 Effect of the intervention strategies on the different health outcomes groups. Each panel shows the total increase in the number of non-FFO visits after the intervention for users belonging to different health risk groups (shades). We classify each individual into different groups of health outcomes depending on which quantile their census tract is with respect to that outcome (darker shading for higher quantiles of each health outcome group). Health outcomes by census tract are obtained from the PLACES Local data for Better Health dataset from the CDC (5). 28
S14 Effect of the mobile food context ϕ in choosing and FFO using the logistic regression Eq. (2) by city as a function of the average value of $\phi(\mathbf{c}_{it})$ by city. Error bars for the mean of ϕ by city are smaller than the symbol size. 29
S15 Total effect of each intervention strategy in each different city. Shades correspond to the number of actions changed by different income quantiles. See Figure 4 in the main text for more information. 30
S16 Effect of the mobile food context in choosing a food outlet at lunchtime for different types of food. Values show the coefficient β (log-odds) in Eq. (2), and bars indicate its standard deviation. The main result in the paper corresponds to the "Fast Food" type. 31
S17 Effect of the mobile phone context in choosing a FFO when going to a food place at lunchtime for different definitions of FFO for different attributions of the stays to the POI and the choice of the stay for the context before lunch. Values show the coefficient β of the logistic regression (log-odds) in Eq. 2) and bars indicate its standard deviation. The result in the main paper corresponds to 200m, within a 1km radius and last stay. 32
S18 Left: Effect of the mobile phone context in choosing a FFO when going to a food place at lunchtime for different definitions of FFO. The analysis is only made for the Los Angeles metropolitan area since the manual definition in (16) is only available there. Values show the coefficient of the logistic regression (log-odds) in Eq. (2), and bars indicate its standard deviation. Right: Number of FO and FFO in the different definitions of fast food outlets. 34
S19 Correlation between home and mobile food environments for different demographic groups. Each group corresponds to a different quartile in each metropolitan area for the % of population with High Education, % of population using public transportation for work, % White population, and median household income. Quartile group 1 correspond to the lowest value and group 4 to the largest value of those demographic traits. 36

List of Supplementary tables

S1 Summary of the mobility data and visits in each metro area in thousands (k) or millions (M). 9
S2 Combinations of other parent categories (not "Food") and categories used to classify venues as food outlets. 11
S3 Results for the Ordinary Least Squares regression of mobile (ϕ_i^m), home (ϕ_i^h) average exposure to FFO environments, and the fraction of FFO visits (μ_i) by individual, as a function of different socio-demographic traits of the census tract where individuals live. The table presents the estimated coefficients with their respective standard errors in parentheses. p-values correspond to two-sided tests for the hypothesis that each coefficient is different from zero. 95% Confidence intervals for R^2 were obtained through bootstrap with 200 realizations. 15
S4 Logistic regression results for the model in Equation (2) for different groups of users and food outings. The last column corresponds to the model in Equation (4) for the DMV visits. The table presents the estimated coefficients with their respective standard errors in parentheses. p-values correspond to two-sided tests for the hypothesis that each coefficient is different from zero. We also report the Squared Correlation and Pseudo R^2 results for the logistic regression, and the Accuracy in the training of the model and its 95% confidence interval in square brackets. 17
S5 Logistic regression results for the model in Equation (2) for food outings happening at different distances from home (d_h). The table presents the estimated coefficients with their respective standard errors in parentheses. p-values correspond to two-sided tests for the hypothesis that each coefficient is different from zero. We also report the Squared Correlation and Pseudo R^2 results for the logistic regression. 18
S6 Logistic regression results for the model in Equation. (3). The table presents the estimated coefficients with their respective standard errors in parentheses. p-values correspond to two-sided tests for the hypothesis that each coefficient is different from zero. Note that d_{it} is the (log-)distance between the context and the food place where lunch happened. 20
S7 Logistic regression results for the model in Equation (2) for all actions throughout the day and different out-of-sample test settings. The table presents the estimated coefficients with their respective standard errors in parentheses. p-values correspond to two-sided tests for the hypothesis that each coefficient is different from zero. We also report the Accuracy in train and test groups and its 95% confidence interval in square brackets. 25
S8 Logistic regression results for the model in Equation (2) for all actions throughout the day and different out-of-sample test settings. The table presents the estimated coefficients with their respective standard errors in parentheses. p-values correspond to two-sided tests for the hypothesis that each coefficient is different from zero. We also report the Accuracy in train and test groups and its 95% confidence interval in square brackets. 33

Supplementary Note 1: Data

The mobility data were obtained from Spectus, a location intelligence, and measurement company. The dataset consists of anonymized records of GPS locations from users who opted-in to share the data anonymously in the US metropolitan areas over a period of 6 months, from October 2016 to March 2017. Data was shared in 2017 under a strict contract with Spectus through their Data for Good program, where they provide access to de-identified and privacy-enhanced mobility data for academic research and humanitarian initiatives only. All researchers were contractually obligated not to share data further or to attempt to de-identify data. Mobility data is derived from users who opted in to share their data anonymously through a General Data Protection Regulation (GDPR) and California Consumer Privacy Act (CCPA) compliant framework.

1.1 Extracting stays

From the data, we extracted the "stays", as the places $\mathbf{x}_{i,t}$ where anonymous user i stayed (stopped) at time t for at least Δt^{roam} minutes using the algorithm proposed by Hariharan and Toyama (14). In that algorithm, a set of consecutive pings are clustered together if their maximum distance from their centroid $\mathbf{x}_{i,t}$ is not larger than some roaming distance Δl^{roam}. The duration of a stay $\tau(\mathbf{x}_{i,t})$ is then the time between the first and last ping belonging to a cluster. Here, as in (22; 16), we use Δl^{roam} = 50 meters and $\Delta t^{roam} = 5$ min. Shorter stays cannot be detected with enough precision by our algorithm. Furthermore, we want to discard people just passing by or stopping for a short time close to the food place. To discard stays related to working places, we only considered stays of less than 2 hours of duration [$\tau(\mathbf{x}_{i,t}) <$ 2 hours]. Only 5.83% of the visits to food outlets are discarded with this last filter (see Figure S1).

1.2 Attribution of stays to POIs

To identify if a stay happens in a particular place, we use a dataset of 1.2 Million Points-of-Interest (POI) in US metropolitan areas collected using the Foursquare API in 2017. We use the Foursquare venue categorization of the places to detect the type of place visited (12). Some of those POIs are classified as Food Outlets (FO) and Fast Food Outlets (FFO); see Supplementary Note Supplementary Note 3:.

Like stays, each POI is also represented by a single point in space. To attribute a stay to a POI, we simply attribute each stay to the closest POI in our dataset. Despite its simplicity, this strategy works well for inferring visited places from sparse, low-accuracy mobile data (8). To avoid attributing a stay to a distant place, we choose only the closest place within a radius of $d^{max} = 200$ meters. However, we find that the median distance of stays attributed to POI is 24.24 m (Interquantil Range, IQR, 12.21 - 49.17), and the median distance of a stay attributed to FO is 18.82 m (IQR, 9.74 - 30.335m) and the median distance of a stay attributed to FFO is 17.70m (IQR, 9.257 - 31.88m). All of them are much smaller than d^{max}. Nevertheless, we have tested that our results are largely independent of and robust to our approach for attributing stays to POI, see Supplementary Note 12.1.

1.3 Identifying home

Finally, we estimate the home Census Block Group of the anonymous users as that in which they are more likely located during nighttime. For each smartphone, we use its stays from 22:00 to 06:00

and spatially cluster them using the Density-based Spatial Clustering of Applications with Boise (DB-SCAN) algorithm (11) to detect the most likely cluster of stays each anonymous user is located in during nighttime and early morning hours. We use 5 as the minimum number of points per cluster and $\varepsilon = 30$ meters as the neighborhood distance in the algorithm. We only consider individuals who were at their home cluster for at least 10 nights in the observation period. The Census Block Group that contains that cluster is identified as the users' home. We also discard stays happening closer than 50 meters from users' home locations.

1.4 Definition of metro areas

We only considered mobility data that happened within 11 metropolitan areas defined as the Core-based Statistical Areas (CBSA) (1), see Table S1. We considered CBSAs instead of other geographical units since they are areas that are socioeconomically related to an urban center. This provides a self-contained metropolitan area in which people move for work, leisure, or other activities. Note that most of the CBSAs we consider span several states.

1.5 Descriptive analysis of the data

Table S1 and Figure S1 show different summaries of the data used in the analysis. In particular, we show the distribution of stay duration for different types of POIs, the distribution of the number of stays per user, and the distributions of μ_i, ϕ_i^m and ϕ_i^h for each individual.

Figure 1B in the main text shows the distributions of the distance traveled to all visits and different food venues. Table S1 shows the number of points in those distributions. The number of points for the grocery stores visits is 1.96 million points. In our dataset, we find that the median distance from home $\mathbf{h}$ to any place visited $\mathbf{x}$ is 7.83km (Interquartile Range, IQR, [2.47km - 18.63km]). For the rest, we found:

- The median distance to any food venue visited is 6.94km (IQR [2.30km - 17.23km]). The Mann Whitney U-Rank test showed that the difference with visits to all places is significant ($p - value < 0.001$ and effect size (rank biserial) $r = 0.03$ with 95% confidence interval $[0.03, 0.03]$).
- The median distance to any fast food venue visited is 6.74km (IQR [2.50km-16.62km]). The Mann Whitney U-Rank test showed that the difference with visits to all places is significant ($p - value < 0.001$ and effect size (rank biserial) $r = 0.02$ with 95% confidence interval $[0.02, 0.03]$).
- The median distance to grocery stores is 3.1km (IQR [1.35km - 8.22km]). The Mann Whitney U-Rank test showed that the difference with visits to all places is significant ($p - value < 0.001$) and effect size (rank biserial) $r = 0.17$ with 95% confidence interval $[0.16, 0.18]$).

More details about how the data was constructed, its representativity, and comparison with other datasets can be found in references (16; 22) and Supplementary Note Supplementary Note 2:. In particular, as found in (22), our sample of users and their behavior is highly representative of the people living in those metropolitan areas, including their socio-demographic profile and the visitation patterns to different amenities. Also, in (16) we found that neighborhood-level features representing visits to fast food outlets (FFO) were significantly associated with self-reported fast-food intake, significantly associated with obesity and diabetes, and were a better predictor of these diseases than self-reported fast food intake.

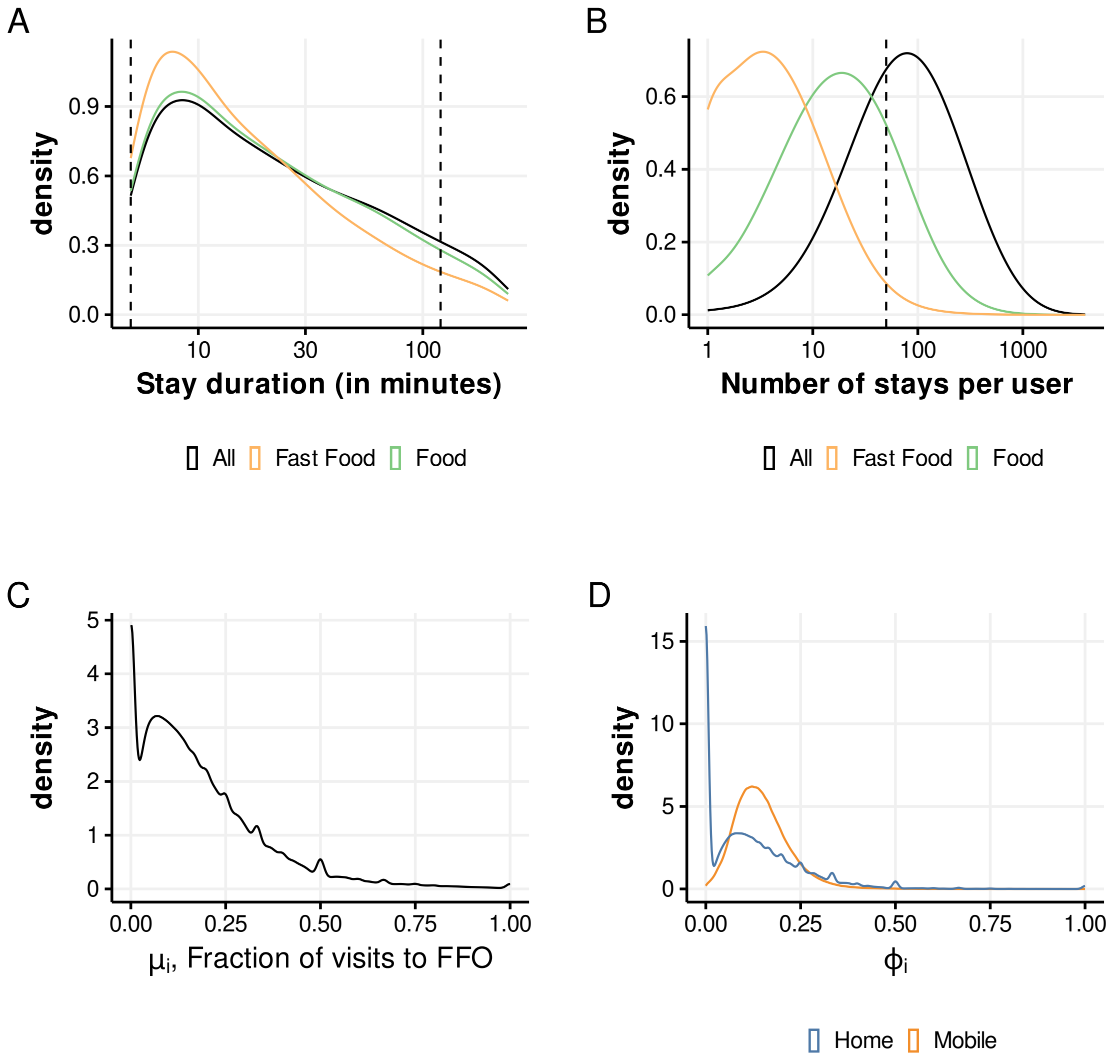

Figure S1: A: Distribution of stay duration (in minutes) in our dataset for all POI, only those attributed to food POI and fast food POI. Vertical lines correspond to the minimum (5 min) and maximum (2 hours) used in our analysis. B: Density of the number of stays per user and type of POI. The vertical line corresponds to 50 stays used in our models for individuals. C: Density of the ratio of FFO to FO visited for each individual. D: Density of the mobile food environments and home environments for each individual.

Table S1: Summary of the mobility data and visits in each metro area in thousands (k) or millions (M).

Metro Area	Number of users	Number of total visits	Number of food visits (% of total visits)	Number of fast food visits (% of total food visits)
Boston	72k	7.49M	2.08M (27.79%)	0.3M (14.45%)
Chicago	300k	39.18M	9.46M (24.14%)	1.85M (19.59%)
Dallas	245k	33.43M	8.13M (24.32%)	1.89M (23.27%)
Detroit	127k	15.91M	3.91M (24.58%)	0.71M (18.16%)
Los Angeles	243k	38.37M	9.92M (25.85%)	1.72M (17.38%)
Miami	187k	26.63M	6.29M (23.63%)	0.93M (14.7%)
New York	248k	35.39M	9.3M (26.26%)	1.19M (12.85%)
Philadelphia	123k	13.68M	3.7M (27.08%)	0.51M (13.84%)
San Francisco	86k	9.6M	2.65M (27.64%)	0.37M (13.98%)
Seattle	73k	8.58M	2.33M (27.16%)	0.43M (18.44%)
Washington	153k	16.77M	4.26M (25.41%)	0.9M (21.01%)
Total	1862k	245.05M	62.04M (25.32%)	10.81M (17.42%)

1.6 Other data used

Demographic characteristics for each Census Block Group were obtained from the American Community Surve (5 years) 2017 edition (28). Each demographic characteristic is in a given table. In our study we used the following demographic characteristics (by CBG):

- Proportion of Black population in the CBG, Table C02003.
- Proportion of employed (civilian population), Table B23025.
- Proportion of people with a college education or beyond, Table B15002.
- Proportion of people working in low-skill jobs, namely in the Agriculture, Construction, Manufacturing, Wholesale, Retail, and Transportation sectors. Table C24030.
- Proportion of people commuting longer than 45 minutes, Table B08303.
- Proportion of people that use public transportation for that commuting, Table B08301.
- Median income of each household, Table B19013.

Census tract-level estimates for different health risk behaviors and outcomes were obtained from the 2017 edition of the PLACES Local data for Better Health dataset from the CDC (5). The data is public and contains prevalence levels of different chronic diseases and health risk behaviors like Diabetes or Physical Inactivity by census tract. Estimations of those prevalence levels are only available for a given set of cities, but it overlaps with the cities in our study.

Finally, Low-Income, Low-Supermarket-Access (LILA) Census tracts were obtained from the 2015 version of the Food Environment Atlas made by the U.S. Department of Agriculture (10). In particular, we consider the LILA tracts cataloged by the U.S. Department of Agriculture as LILA 1-and-10 (flag for food desert), which are those that have low-income and low accessibility to supermarkets measured at 1 mile for urban areas and 10 miles for rural areas.

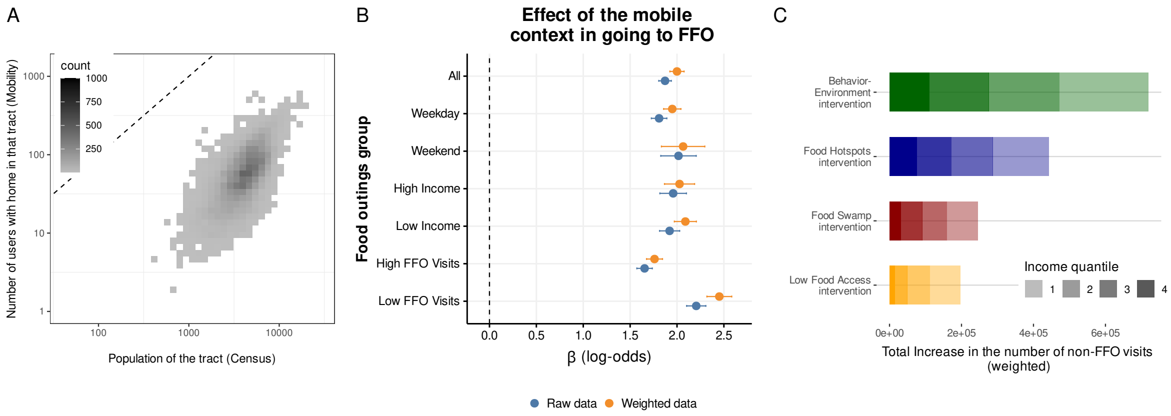

Figure S2: A) Correlation between the smartphone population detection in our mobility data and the official census population by census tract. B) Coefficients of the logistic regression coefficient without (raw) and with (weighted) weights in the regression proportional to $1/w_{\Omega_i}$. C: Effect of the different interventions re-weighted by $1/\Omega_i$ to account for the different ratio of users to the population in each census tract Ω.

Supplementary Note 2: Representativity

Our location data comes from smartphones in large urban areas. Although a large proportion of the U.S. population owns a smartphone in urban areas, we might question whether our sample of 1.8 million devices is representative of the population and different socio-demographic groups in that area. Figure S2 shows the comparison between the population detected in our data and the census data. As we can see, the correlation is high ($\rho = 0.59 \pm 0.01$) by census tract, showing that we get a good representation of the population. Despite that, similar to reference (22) we address the representativity of the data using a weighting mechanism (post-stratification) based on w_{Ω_i}, the ratio of smartphone users to the true population in census tract Ω_i where user i lives (26). We tested that our main results do not depend on this post-stratification technique. In particular, as we can see in figure S2b if we weigh the data in the logistic regression model (2) by the inverse of the ratio, we get very similar results to the raw data. Still, the impact on the environment is of the same magnitude as the main results presented in the paper. Similar results are obtained when we re-weigh the effect of different interventions by the inverse of the ratio of smartphone users to the true population. In Figure S2c, we compare the effect of different interventions on our sample of users, compared with the weighted version:

$$\Delta^{\mathrm{FFO}}(\Omega) \simeq \sum_{\mathbf{c}_{it} \in \Omega} \frac{\beta}{w_{\Omega_i}} \frac{e^{X_{it}}}{(1+e^{X_{it}})^2} \frac{\delta \phi}{\delta \mathrm{I}}.$$

As we can see, the relative results between the different interventions are similar between the weighted and non-weighted (raw) data. But obviously, the numbers are scaled to population levels.

Table S2: Combinations of other parent categories (not "Food") and categories used to classify venues as food outlets.

Parent Category	Category
Nightlife Spot	Sports Bar, Bar, Pub, Dive Bar, Speakeasy, Hookah Bar, Lounge, Seafood, American, Cocktail, Italian, Brewery, Beer Garden, Hotel Bar, Wine Bar, Mediterranean, Restaurant, Whisky Bar, Mexican, New American, Tiki Bar, Sake Bar, Liquor Store, Pizza, Gastropub, Wings, Sushi, Steakhouse, Coffee Shop, Korean, Wine Shop, Burgers, BBQ, Winery, Café, Beer Bar, French, Beach Bar, Sandwiches, Japanese, Asian, Convenience Store, Piano Bar, Beer Store, Bakery, Grocery Store, Desserts, Gourmet, Deli / Bodega, Juice Bar, Food Truck, Candy Store, Food & Drink, Cafeteria, Butcher, Snacks, Cheese Shop, Corporate Cafeteria, Supermarket, Street Food Gathering
Shop & Service	Grocery Store, Convenience Store, Liquor Store, Butcher, Gourmet, Food & Drink, Health Food Store, Warehouse Store, Discount Store, Deli / Bodega, Fish Market, Wine Shop, Candy Store, Beer Store, Cheese Shop, Supermarket, Sports Bar, Coffee Shop, Sandwiches, Farmer's Market, Snacks, Seafood, Fruit & Vegetable Store, Chocolate Shop, Café, American, Italian, Restaurant, Desserts, Gastropub, Bakery, Juice Bar, Herbs & Spices Store, Pizza, Winery, Dive Bar, Steakhouse, Food Truck, Burgers, Beer Garden, Lounge, Smoothie Shop, New American, Street Food Gathering, Organic Grocery, Wine Bar, Korean, Bar, Hookah Bar, Pub, Japanese, Mexican, Cocktail, Brewery, Sushi, Asian, Mediterranean, Speakeasy, Beer Bar, Tiki Bar, Cafeteria, BBQ, Whisky Bar, French, Hotel Bar, Sake Bar
Arts & Entertainment	Bar, Italian, Mexican, Piano Bar, Fish Market, Coffee Shop, Pub, American, New American, Restaurant, Gastropub, Sports Bar, Farmer's Market, Pizza, Lounge, Dive Bar, BBQ, Café, Steakhouse, Sushi, Wine Bar, Mediterranean, Desserts, Food Truck, Brewery, Beer Garden, Juice Bar, Cocktail, Speakeasy, Candy Store, Beach Bar, Burgers, Snacks, Bakery, Cafeteria, Japanese, Grocery Store, Food & Drink, Winery, Whisky Bar, Hotel Bar, French, Seafood, Asian, Wine Shop, Sake Bar, Hookah Bar, Beer Bar, Deli / Bodega, Sandwiches, Liquor Store, Cheese Shop, Convenience Store
Professional & Other Places	Winery, Liquor Store, Lounge, Coffee Shop, French, Italian, Corporate Cafeteria, Wine Shop, Hotel Bar, Chocolate Shop, American, Farmer's Market, Speakeasy, Bar, Fruit & Vegetable Store, Corporate Coffee Shop, Seafood, Café, Burgers, Cafeteria, Convenience Store, Grocery Store, Asian, Food & Drink, Deli / Bodega, Pub, Bakery, Restaurant, Candy Store, Mexican, Wine Bar, Whisky Bar, Sports Bar, Warehouse Store, Cocktail, Pizza, BBQ, Brewery, Mediterranean, Desserts, Hookah Bar, Beer Garden, Discount Store, Health Food Store, Snacks, Sandwiches, Food Truck, Japanese, Supermarket, Dive Bar, Steakhouse, New American, Gourmet, Juice Bar, Beer Bar, Cheese Shop, Beer Store
Travel & Transport	Lounge, Food Truck, Bar, Hotel Bar, Cocktail, Deli / Bodega, American, Pizza, Brewery, Restaurant, Cheese Shop, Pub, Bakery, Beer Garden, New American, Mediterranean, BBQ, Café, Liquor Store, Sports Bar, Sushi, Mexican, Convenience Store, Burgers, Wine Bar, Coffee Shop, Dive Bar, Beach Bar, Speakeasy, Italian, Seafood, Steakhouse, Wings, Sandwiches, Winery, Gastropub, French, Grocery Store, Korean
College & University	Cafeteria, Bar, Coffee Shop, Italian, Convenience Store, Café, Food Truck, American, Burgers, Restaurant, Pub, Speakeasy, Gourmet, Deli / Bodega, Sandwiches, Dive Bar, Cocktail, Snacks, Wine Bar, Hookah Bar, Lounge, BBQ, Asian, Candy Store, Sushi, Pizza, Sports Bar, Chocolate Shop, Juice Bar, French, Grocery Store, Bakery, Food & Drink
Outdoors & Recreation	Food Truck, Fish Market, American, Juice Bar, Farmer's Market, Liquor Store, Food & Drink, Italian, Winery, Grocery Store, Seafood, Lounge, Beer Garden, Sandwiches, Wine Bar, Sports Bar, Bar, Restaurant, Hotel Bar, Coffee Shop, Deli / Bodega, Street Food Gathering, Café, Burgers, Health Food Store, Mexican, Bakery, Speakeasy, Hookah Bar, Pub, Snacks, Cocktail, Brewery, Dive Bar, New American, Pizza, French, Butcher, Beach Bar, Asian, Supermarket, BBQ, Gastropub, Convenience Store, Discount Store, Corporate Cafeteria
Residence	Speakeasy, Beer Garden, Lounge, Food & Drink, Wine Bar, Deli / Bodega, Grocery Store, Café, Brewery, Coffee Shop, Cocktail, BBQ, Hotel Bar, Whisky Bar, Gourmet, American, Beach Bar, Pizza, Mediterranean, Bar

Supplementary Note 3: Classification of Food Outlets

Food visits were defined as visits to a location where food might be sold (including restaurants, food retailers, and other locations). We extend the idea in (7) and identify Food Outlets (FO) using a combination of Foursquare's existing categorization taxonomy (12). Each place in the Foursquare dataset belongs to a parent category (e.g., "Food" or "Arts & Entertainment"). In particular, FO are defined as those in the "Food“ parent category, but we also include other places that serve or sell food that happen in other parent categories, probably due to misclassifications or FO within multipurpose facilities (e.g., music venues that serves food). A detailed description of those combinations appears in Table S2. For privacy reasons, we have excluded FO located at schools, drug stores, or medical facilities.

To identify Fast Food Outlets (FFO) we use the name of the venue and match it to the list of Fast Food restaurant chains in the US from the Wikipedia (33). We augmented that list to get common

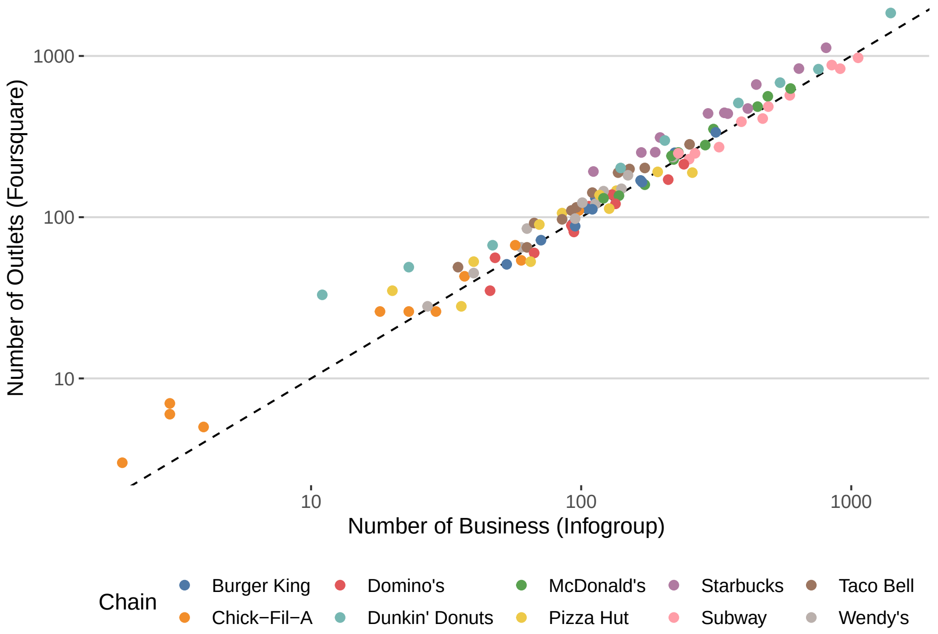

Figure S3: Comparison between the number of FFO by chain in different cities obtained from our Foursquare dataset and Infogroup's US Historical Business Data for 2016 (17).

names like "KFC" instead of the official "Kentucky Fried Chicken". And to account for potential different spellings of the name (e.g. "Carl's Jr" instead of "Carl's Jr. / Green Burrito") we used approximate string matching between the name of the venue and the list in the Wikipedia using the Jaro-Winkler distance (6) of the `stringdist` library (29). To test how good is this classification of FFO, we compare it with the manual annotation of FFO for the LA metro area in (16), which used a combination of Foursquare's existing FFO categorization taxonomy and a bottom-up search of known chain FF outlet names validated in previous research (9) and string matching techniques. We found that 94% of the places annotated as FFO in (16) were included in our automatic classification. However, our classification contains more FFO (6964) than those in (16) (4151) in LA, because some major chains like "Chipotle" or "Starbucks" were not considered FFO in (16). In Section Supplementary Note 12: we check extensively that our results do not depend on the definition and set of FFO considered.

Finally, our Foursquare dataset is an accurate representation of the fast-food outlets in each city. As we can see in Figure S3, the number of FFO detected in each city by fast-food chains has a strong correlation with other (curated) datasets of business, like the Infogroup's US Historical Business Data for 2016 (17). The small variation between them can be attributed to the different ways an outlet is defined in both datasets. Also, in our dataset, we can detect food outlets within businesses (e.g., coffee shops in large stores) which do not appear in Infogroup's dataset.

Supplementary Note 4: Definition of context and food visit

To understand the role of individual preferences and the context where food decisions are made, we restrict the analysis to visits to FO during lunch hours (from 11h30 to 14h local time) because they are more frequent, more likely to involve a visit to a venue for food, but most importantly, because they are more likely to be affected by the local context where individuals were previously. As we can see in Figure 2 in the main text, visits to FO and FFO are more frequent around lunchtime. For those food decisions y_{it} at time t, we take the last place in the morning (until 11h30) where users were observed (context $\mathbf{c}_{it}$), and characterize the context where that decision is made by $\phi(\mathbf{c}_{it})$. To make sure that there was a possible decision between FFO and non-FFO options, we only keep observations where the context $\mathbf{c}_{it}$ contains both types of places. After those restrictions, we got 2.7 million visits to FO during lunchtime by 755k users.

Supplementary Note 5: Detecting change of context

To detect those users that change their context before lunch, we used the following method: we take the sequence of the census tracts where c_{it} is. We only take those users that have at least two different census tract contexts with more than 30% of their total visited contexts during that time period, and we consider only users that appear at least five times at each of those two tracts. With only those two census tract contexts, we construct the binary time series between them; see Figure S4. We apply a method to detect if and where a changepoint occurred in that time series based on detecting statistical changes in the mean using the (18). That method was implemented using the library `changepoint` in `R`, and we use a manual penalty using n^γ for the number of changepoints n. We manually annotated some cases and found that $\gamma = 0.1$ yielded the best accuracy in detecting changes in contexts. If the method detects a changepoint, the properties of the mobile food environment before and after it are averaged over all $\mathbf{c}_{it}$ in each of the census tracts' contexts. Using this method, we detected 8524 users in our dataset that changed contexts during our observation period.

Supplementary Note 6: Estimation of the effect of changing contexts

To provide a statistically robust estimation of the impact of those individuals that changed their context before lunch, we design a methodology based on the Bayesian Structural Time Series (BSTS) approach. We define four groups of users depending on whether their contexts before/after has Low ($\phi < 0.13$) or High ($\phi > 0.13$) (average) ratio of fast food options. Around 35% of users changed from Low $\to$ Low and another 35% from High $\to$ High, while 15% changed from Low $\to$ High and another 15% from High $\to$ Low. To study the effect of changing from Low $\to$ High, for example, we consider the time series $\mu_t^{\text{Low}\to\text{High}}$ of the fraction of FFO visits at lunch by day t, where $t = 0$ is when they changed their context. As a counterfactual, we consider those users that changed their context also, but changed to a similar number of FFO options contexts, $\mu_t^{\text{Low}\to\text{Low}}$. Note that we did not use as counterfactual those users that stayed in the same geographical context, but only those that changed their geographical context. This was done to reduce the possibility of some endogeneity between changing contexts and the food environment in the previous context. Similarly to study the effect of changing from High $\to$ Low contexts we use $\mu_t^{\text{High}\to\text{Low}}$ taking $\mu_t^{\text{High}\to\text{High}}$ as counterfactual. BSTS models were implemented using the `CausalImpact` packages in `R` (4). Given a response and a counterfactual time series, BSTS predicts how the response would have evolved after the change to

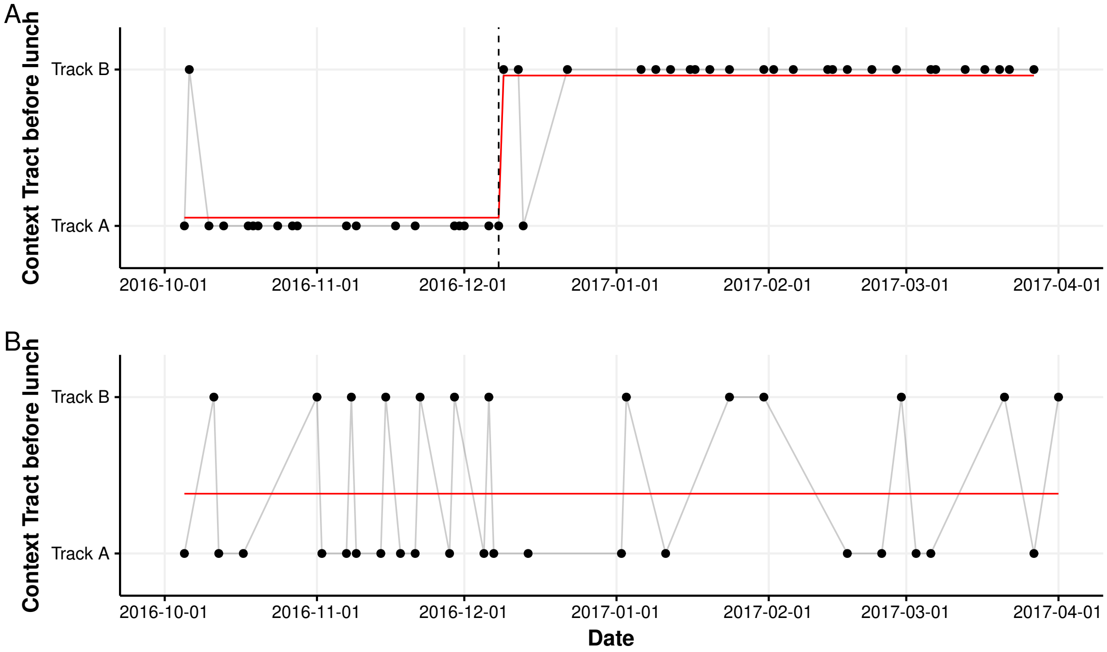

Figure S4: Method to detect the change of context before lunch. We construct a binary time series for each of the census tracts for those users that have more than 30% of their contexts in two of them. We use a statistical test to detect statistically significant changes in the mean of those time series to know if and where changepoints happened. For the user in panel A, a significant change in the means (in red) is detected in December. However, for the user in panel B, no significant change is detected, and the user changes often between contexts. Statistically significant changes were detected using the `changepoint` library with a manual method and penalty of $n^{1/10}$ (see details of those methods in (18)).

a different context if the change never happened. In the modeling, we used the default values of the `CausalImpact` library, including no seasonality, since our time series are centered 50 days around where they change their context.

Supplementary Note 7: Models

7.1 Demographic models for exposure to home and mobile food environments and FFO visits

To investigate the socio-demographic differences in home and mobile exposure to fast food, and in the fraction of FFO visit decisions, we have used simple linear regression models like:

$$\phi_i^m, \phi_i^h, \mu_i \sim \sum_l \beta_l d_{l,i} + \mathrm{MSA}_i \tag{1}$$

where $d_{l,i}$ are the different socio-demographic variables associated with the home Census Block Group (CBG) where user i lives, and MSA_i is a fixed factor by urban area. The socio-demographic variables considered are listed in Section Supplementary Note 1:. To prevent large fluctuations in the fraction of

Table S3: Results for the Ordinary Least Squares regression of mobile (ϕ_i^m), home (ϕ_i^h) average exposure to FFO environments, and the fraction of FFO visits (μ_i) by individual, as a function of different socio-demographic traits of the census tract where individuals live. The table presents the estimated coefficients with their respective standard errors in parentheses. p-values correspond to two-sided tests for the hypothesis that each coefficient is different from zero. 95% Confidence intervals for R^2 were obtained through bootstrap with 200 realizations.

	Dependent variable:		
	ϕ_i^m	ϕ_i^h	μ_i
Median Income	0.002 (0.0002)	−0.019 (0.0004)	−0.0002 (0.0005)
	$p < 0.001$	$p < 0.001$	$p = 0.722$
Proportion of Black	0.022 (0.0004)	0.048 (0.001)	0.037 (0.001)
	$p < 0.001$	$p < 0.001$	$p < 0.001$
Proportion of high education	−0.016 (0.001)	0.025 (0.001)	−0.027 (0.001)
	$p < 0.001$	$p < 0.001$	$p < 0.001$
Proportion employed	0.007 (0.001)	0.027 (0.003)	0.005 (0.003)
	$p < 0.001$	$p < 0.001$	$p = 0.106$
Proportion using public transportation	−0.093 (0.001)	0.019 (0.001)	−0.106 (0.001)
	$p < 0.001$	$p < 0.001$	$p < 0.001$
Proportion with long commutes	0.050 (0.001)	−0.073 (0.001)	0.068 (0.001)
	$p < 0.001$	$p < 0.001$	$p < 0.001$
Proportion employed in low skill jobs	0.040 (0.001)	0.008 (0.001)	0.043 (0.002)
	$p < 0.001$	$p < 0.001$	$p < 0.001$
Constant	0.098 (0.002)	0.262 (0.004)	0.165 (0.005)
	$p < 0.001$	$p < 0.001$	$p < 0.001$
Fixed effects by urban area	YES	YES	YES
Observations	1,001,733	1,001,733	1,001,733
R^2	0.213	0.038	0.052
Adjusted R^2	0.213	0.038	0.052
Confidence Interval for R^2	[0.2113,2143]	[0.0374,0.0391]	[0.0513,0.0530]
Residual Std. Error (df = 1001715)	0.063	0.128	0.155
F Statistic (df = 17; 1001715)	15,921.330***	2,342.803***	3,240.151***
Note:			*p<0.1; **p<0.05; ***p<0.01

FFO visits or exposure due to small numbers in the number of visits, in our models we only consider users with more than 50 stays (see Figure S1). Table S3 shows the results of the linear regression for each variable. As we can see, despite many of the coefficients being significant, only the model for ϕ^m has some explanatory power with $R^2 = 0.21$. Interestingly, the fraction of FFO visits and exposure at home models do have a very small explanatory variable.

Note also that, as mentioned in the main text, using bootstrap methods we can see that the difference between the models for ϕ_i^m and ϕ_i^h is statistically significant at 95% level.

Note that our models are multivariate. Thus the correct interpretation of the results in Table S3 is that the coefficient estimate for each demographic variable measures the association of that demographic variable conditioned on the rest of the variables kept constant. However, demographic variables are not independent, and that cannot be done without affecting the variable itself. As we see in Figure S5, demographic variables like income, education, employment, or low-skill jobs for

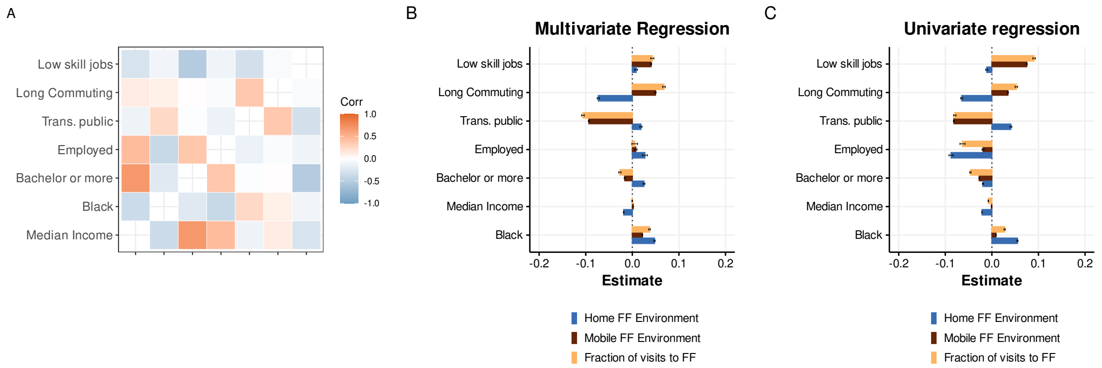

Figure S5: A) Correlogram between the different demographic variables used in our regressions. B) Result of the multivariate regression for the home and mobile food environments and the fraction of visits to FFO fitting a model using all demographic variables, see Eq. (2). C) Result of the univariate regression for the home and mobile food environments and the fraction of visits to FFO using only a given demographic variable (see text).

each CBG are moderately correlated. For that reason, results for a single univariate model like $\phi_i^m, \phi_i^h, \mu_i \sim \beta_l d_{l,i} + \mathrm{MSA}_i$ are different for some of those variables, see Figure S5. Especially significant are cases like the relationship between educational attainment and the fraction of FFO in neighborhoods. As we can see, the univariate relationship is negative (more educational attainment in the neighborhood is related to less FFO in that neighborhood), as expected. However, when we perform the multivariate regression (1), we get that the coefficient is positive, a result of the complex correlational structure of the variables mentioned before.

We have also tested the possibility that demographic variables have better explanatory power using non-linear models to explain home and mobile exposure to fast food. To this end, we have implemented random forest models similar to Eq. (1). Since many users share the same ϕ_i^h for the same demographic variables, to prevent overfitting in that case we have trained the model for ϕ_i^h using a set of home census tracts different than the ones used in testing the model. Our results for non-linear regressions are very similar to the linear ones: we get $R^2 = 0.288$ for ϕ_i^m, $R^2 = 0.048$ for ϕ_i^h, and $R^2 = 0.055$ for μ_i (compare to those in Table S3). Although performing slightly better than linear models, our results show that demographic variables have small explanatory power for both the home and mobile environments. More importantly, more sophisticated models show the small dependence of visits to FFO (μ_i) with demographic variables.

7.2 Model for visits to FO during Lunchtime

To test for the effect of FFO environments, we have run a number of statistical models. For the main results of the paper, we establish the effect of FFO environments around the contexts to go to a FFO place using logistic regression for the probability that the user i went to a FFO at time t, i.e., $y_{it} = 1$.

$$\Pr(y_{it} = 1) = \mathrm{logit}^{-1}[\beta_0 + \alpha_i + \delta_t + \beta\phi(\mathbf{c}_{it})], \quad (2)$$

where $\mathrm{logit}^{-1}(x) = e^x/(1+e^x)$, and $\phi(\mathbf{c}_{it})$ is the fraction of FFO options around the context before lunch. We control individual preferences and daily patterns by introducing a fixed effect by user (α_i)

and day (δ_t). Regression was only performed for those individuals or days that have at least one FFO and non-FFO visit. Results for this model are presented in Figure 2 of the main paper and Table S4. We also cluster errors by day to account for potential heterogeneity; see Table S4.

Table S4: Logistic regression results for the model in Equation (2) for different groups of users and food outings. The last column corresponds to the model in Equation (4) for the DMV visits. The table presents the estimated coefficients with their respective standard errors in parentheses. p-values correspond to two-sided tests for the hypothesis that each coefficient is different from zero. We also report the Squared Correlation and Pseudo R^2 results for the logistic regression, and the Accuracy in the training of the model and its 95% confidence interval in square brackets.

Dependent Variable:	y_{it}							
Food Outings group:	All	Weekdays	Weekends	Income Q1	Income Q4	Low FFO	High FFO	DMV
Variables								
$\phi(\mathbf{c}_{it})$	1.872	1.809	2.016	1.921	1.959	2.205	1.654	1.086
	(0.0335)	(0.0413)	(0.0946)	(0.0536)	(0.0709)	(0.0512)	(0.0413)	(0.1974)
	$p < 0.001$	$p < 0.001$	$p < 0.001$	$p < 0.001$	$p < 0.001$	$p < 0.001$	$p < 0.001$	$p < 0.001$
$\hat{\alpha}_i$								5.065
								(0.1298)
								$p < 0.001$
Intercept								-2.695
								(0.0464)
								$p < 0.0001$
Fixed-effects								
day	Yes	Yes	Yes	Yes	Yes	Yes	Yes	
user	Yes	Yes	Yes	Yes	Yes	Yes	Yes	
Fit statistics								
Observations	1,483,379	1,032,172	182,487	458,358	283,765	789,554	697,838	18,733
Squared Correlation	0.18820	0.19419	0.13372	0.19326	0.18070	0.12651	0.16814	0.10441
Pseudo R^2	0.16054	0.16436	0.10645	0.16521	0.15396	0.12179	0.13335	0.10151
Accuracy (Train)	0.7546	0.7483	0.6831	0.7584	0.7531	0.8090	0.6936	0.8201
Accuracy 95% CI	[0.7539, 0.7553]	[0.7474, 0.7491]	[0.681, 0.6853]	[0.7572, 0.7597]	[0.7515, 0.7547]	[0.8081, 0.8098]	[0.6925, 0.6946]	[0.8145, 0.8255]

*Signif. Codes: ***: 0.01, **: 0.05, *: 0.1*

To observe potential non-linearities in the effect of FFO, we used a logistic regression where we one-hot encoded the rounded version of $\phi(\mathbf{c}_{it})$. This allows us to observe whether the effect they have is linear, or has another shape. The coefficient for each value of ϕ can be seen in figure S7. The effect is always increasing, and it is almost linear, as we hypothesize in Equation (2).

We have also checked the performance of the model at different distances from home. As we can see in Table S5, we still see a strong impact of the context in the decisions to visit FFO. Note, however, that as decisions are made close to home, the impact of the mobile food environment is smaller. This reflects slightly different consumption behaviors at home and on the move. However, as we can also see, most of the food outings happen away from home, where the context is more important to make the decision to visit FFO or not.

In our model, we have considered decisions y_{it}, which are done away from the pre-lunch context.

Table S5: Logistic regression results for the model in Equation (2) for food outings happening at different distances from home (d_h). The table presents the estimated coefficients with their respective standard errors in parentheses. p-values correspond to two-sided tests for the hypothesis that each coefficient is different from zero. We also report the Squared Correlation and Pseudo R^2 results for the logistic regression.

Dependent Variable:				y_{it}			
Model:	$d_h < 2$km	$d_h < 4$km	$d_h < 8$km	$d_h < 16$km	$d_h < 32$km	$d_h < 64$km	All
Variables							
env_context_FFO	0.9487	1.153	1.338	1.496	1.637	1.834	1.872
	(0.2457)	(0.1129)	(0.0666)	(0.0477)	(0.0412)	(0.0338)	(0.0335)
	$p < 0.001$	$p < 0.001$	$p < 0.001$	$p < 0.001$	$p < 0.001$	$p < 0.001$	$p < 0.001$
Fixed-effects							
day	Yes	Yes	Yes	Yes	Yes	Yes	Yes
user	Yes	Yes	Yes	Yes	Yes	Yes	Yes
Fit statistics							
Observations	126,597	254,220	490,368	846,534	1,081,639	1,443,584	1,483,379
Squared Correlation	0.18739	0.18720	0.18793	0.18972	0.19014	0.18868	0.18820
Pseudo R^2	0.15608	0.15658	0.15808	0.16053	0.16148	0.16089	0.16054
BIC	440,169.3	883,018.6	1,646,485.5	2,698,369.4	3,354,818.0	4,338,259.9	4,438,285.7

Clustered (day & user) standard-errors in parentheses
*Signif. Codes: ***: 0.01, **: 0.05, *: 0.1*

For our lunchtime dataset, the distance from home to the FO visit has a median of 9.78km, while the median from the context is only 1.60km. Despite that, we get a number of actions y_{it} that happen far away from the context. One important question is how the context influences those decisions that are taken far away; that is, what is the role of the distance between the context of action in the food choice? This could be entangled with the question of how food environments are spatially correlated. If $\phi(x)$ has a large spatial correlation, we might find that the context is still important to explain decisions taken far away from it. However, as figure S6 shows, the spatial correlation of $\phi(x)$ decays dramatically after 1km. Thus the food environments where the decision and action are made are very different in terms of FFO options.

To understand better the role of distance in the effect of the context, we have modified the model (2) to include the distance as a modulating factor in the effect of the food environment:

$$\Pr(y_{it} = 1) = \text{logit}^{-1}[\beta_0 + \alpha_i + \delta_t + \log d_{it} \times \beta\phi(\mathbf{c}_{it})], \tag{3}$$

where $\log d_{it}$ is the (log-)distance between the context before lunch $\mathbf{c}_{it}$ and the food outlet where lunch happened. Results are presented in table S6, where we can still see that the effect of the context is strong $\beta = 3.335$ (0.0541) but is modulated by the distance. The interaction effect is negative meaning that, as expected, for larger distances the impact of the context is smaller, and it could be even negative for larger distances. In particular, we find that the effective log-odds become negative when $d_{it} > \exp(3.335/2.091) \simeq 4.93km$ (see Figure S6B). This might be because people who travel such large distances between context and action probably go to places that are very different from the context (see Figure S6A). In any case, the heterogeneity of the coefficients at different distances should be viewed as merely correlational, meaning that when people travel far from their context to go for lunch, their meal decision is less correlated with their context than when they travel less.

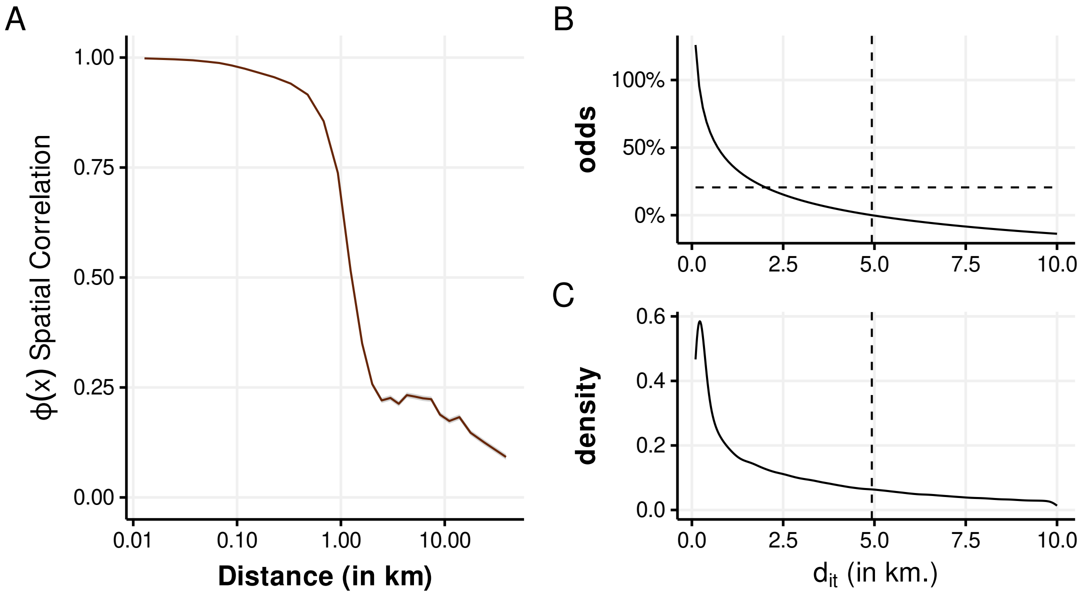

Figure S6: A: Spatial autocorrelation of $\phi(x)$, as a function of the distance in km. B: change in odds of visit to an FFO at different distances between the context and the lunch action when the context includes 10% more FFO, obtained from the results in Table S6. C: Distribution of the distance between the context and the lunch action. The vertical dashed line corresponds to $d_{it} = \exp(3.335/2.091)$, see Table S6, and the horizontal line in B corresponds to the value of the model in the main text for all distances ($\sim$ 20%).

However, for the majority of our data, the distance d_{it} is smaller than 5km (only 15% of it has $d_{it} > 4.93km.$, see Figure S6C). Thus, for a large majority of the actions in our dataset, we still get that the impact of the context is positive: more FFO in the context increases the probability that the lunch action is a visit to an FFO.

7.3 Model for visits to FO by socio-demographic traits

We have also tested whether the effect of the context could be accounted for the different contexts that individuals visit depending on their socio-demographic traits. As we can see in Figure 2 in the main paper, people that rely more on public transportation and long commuting, with low-skill jobs, and from predominantly Black neighborhoods seem to be exposed to more FFO. However, as we see in Figure 2 and Figure 3 in the main paper, the FFO context and its effect seem to be largely independent of income. Rather than introducing complicated interaction terms, to test other demographic variables in the effect of the context, we have rerun the logistic regressions only for the visits to FO of particular socio-demographic groups, specifically by quantile of the different socio-demographic variables. As we can see in Figure S7, although there is some variability, our estimations of β are very similar across different socio-demographic groups.

Table S6: Logistic regression results for the model in Equation. (3). The table presents the estimated coefficients with their respective standard errors in parentheses. p-values correspond to two-sided tests for the hypothesis that each coefficient is different from zero. Note that d_{it} is the (log-)distance between the context and the food place where lunch happened.

Dependent Variable:	y_{it}
Variables	
$\phi(\mathbf{c}_{it})$	3.335*** (0.0541), $p < 0.001$
$\log d_{it}$	0.3668*** (0.0070), $p < 0.001$
$\log d_{it} \times \phi(\mathbf{c}_{it})$	-2.091*** (0.0329), $p < 0.001$
Fixed-effects	
Day	Yes
User	Yes
Fit statistics	
Observations	828,644
Squared Correlation	0.18163
Pseudo R^2	0.15187
BIC	2,792,925.6

Clustered (day & user) standard-errors in parentheses

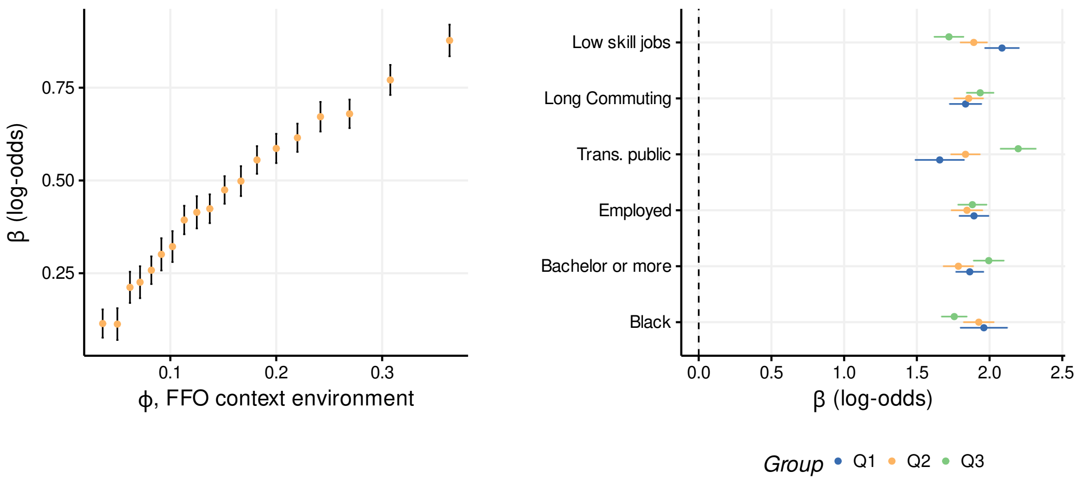

Figure S7: Left: Effect of the mobile food context ϕ in choosing an FFO using logistic regression and hot-encoding different values of ϕ. Error bars are standard errors of the coefficients. Right: Effect of the mobile food context in choosing an FFO restricted to the food outings of different socio-demographic groups. Groups are selected as quantiles of each socio-demographic variable.

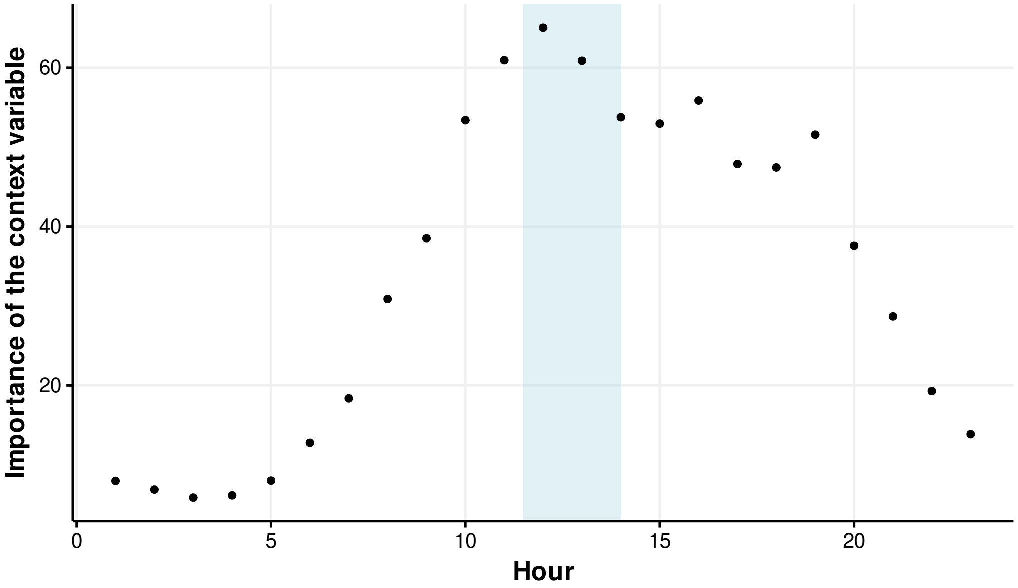

Figure S8: Importance of the FFO context variable $\phi(\mathbf{c}_{it})$ for models built with actions at different hours of the data. Variable importance is given by the absolute value of the t-statistic of the variable in the regression model (2) by the hour.

7.4 Model for the visits to FO after DMV

In the case of visits to DMV, we typically have only one observation and day per user. Thus we have removed the daily and individual fixed factors and we have used a simpler logistic regression model:

$$\Pr(y_{it}^{\mathrm{DMV}} = 1) = \mathrm{logit}^{-1}[\hat{\alpha}_i + \beta\phi(\mathbf{c}_{it})] \tag{4}$$

where $\hat{\alpha}_i$ is the observed fraction of visits to FFO for each individual i.

7.5 Model for the visits to FO during the whole day

Finally, we have extended the model (2) to the rest of the day. To this end, we consider each food visit and find the previous (non-food) stay within two hours in the past as the context $\mathbf{c}_{it}$. Then we fit a similar model as that in Eq. (2). This allows us to investigate also at what time of the day the context is more important to determine the type of food visit. As we can see in Figure S8, the importance of the context variable $\phi(\mathbf{c}_{it})$ measured by the t-statistic in the regression model (2) is larger around noon. Thus, visits around noon are more likely to be affected by the local context where individuals were previously.

Since this model for the whole day is used in our estimation of the effect of interventions, we have also investigated its performance in predicting users' behavior. Table S7 shows the accuracy of the logistic regression model during training and testing under different out-of-sample situations.

- Individual out-of-sample: In the first analysis, we considered the usual out-of-sample test in which a random fraction of actions were removed when training the model and used later to measure model performance for unseeing actions.

- Geographical out-of-sample: We have also considered a geographic out-of-sample test in which we trained the model only on the actions in a fraction of census tracts and used the actions in the remaining census tracts as a test set. This way, the model was trained without incorporating any information about the environments in census tracts in the test group. That is, we test then the performance of the model when users are subject to unseen or different environments, a situation similar to our interventions.

In both cases, we have chosen 75% for the fraction in the train set and 25% for the test set. As we can see in Table S7 the model achieves high performances (accuracies in the test set around 80%) in both cases at the level of individual actions.

But since our analysis of interventions is based on the total number of visits to FFO by area, our results critically depend on the performance of the model at the level area. To evaluate that performance by area we have compared the predictions of our model for both out-of-sample strategies on the total number and fraction of FFO visits by area. The results in Figure S9 show that the model achieves highly accurate predictions. Of special importance is the accuracy in the geographical out-of-sample: training the model in some areas is able to capture both the individual preferences and the effect of the food environment so well that when we use the model to predict the visits to FFO in totally different areas, the correlation between the real data and our predictions is around 95% for the total number of visits to FFO and around 70% for the fraction of FFO.

Although our logistic regression model is quite accurate, we have tried other non-linear models (like Random Forest) to see if they have better performance. However, we get worse prediction accuracy (around 60% for random forest) compared with logistic regression. This is expected, given that random forest regressions are worse than logistic ones when the number of variables used as regressors is small (20). Also, as we see in Figure S7, the effect of the context is almost linear with $\phi(\mathbf{c}_{it})$, something which is already accounted for in linear models like the logistic regression.

In summary, our logistic regression model, Equation (2), has high accuracy in predicting visits to FFO at individual and, more importantly, at a given geographical area.

Supplementary Note 8: Topic analysis of census tracts

To identify the type of areas where the most efficient interventions happen, we have used topic modeling to identify the different patterns behind the distribution of POIs by area. We note that mobility data or visits were not used in this analysis, only the spatial distribution of POIs from the Foursquare API. Similar to LDA applied to text classification, we describe every census tract by the number of POIs in each category (terms), and we have applied Latent Dirichlet allocation (LDA) to get a small number of groups of POIs (topics) to describe each census tract (document) (3). For our analysis, we have only considered those categories that appear at least in 20 or more census tracts, so we ended up with a document-term matrix of 18898 census tracts with 614 categories of POIs.

The result of the LDA applied to that document-term matrix are two matrices: one containing the weights of the different categories by topics and the other containing the probabilities of finding a specific topic within each census tract. To find the number of topics, we have used different metrics found in the `ldatuning` package (23). Results for this tuning are found in figure S10 where we can see that we get (almost) global minima and maxima for different metrics around $k = 20$.

The composition of the different topics can be found in figure S11. As we can see, the topics are easily recognizable, and we have given names to each of them for easy recognition. They go from groups of POIs related to airports, offices, industrial / factory areas, shopping centers, malls, health

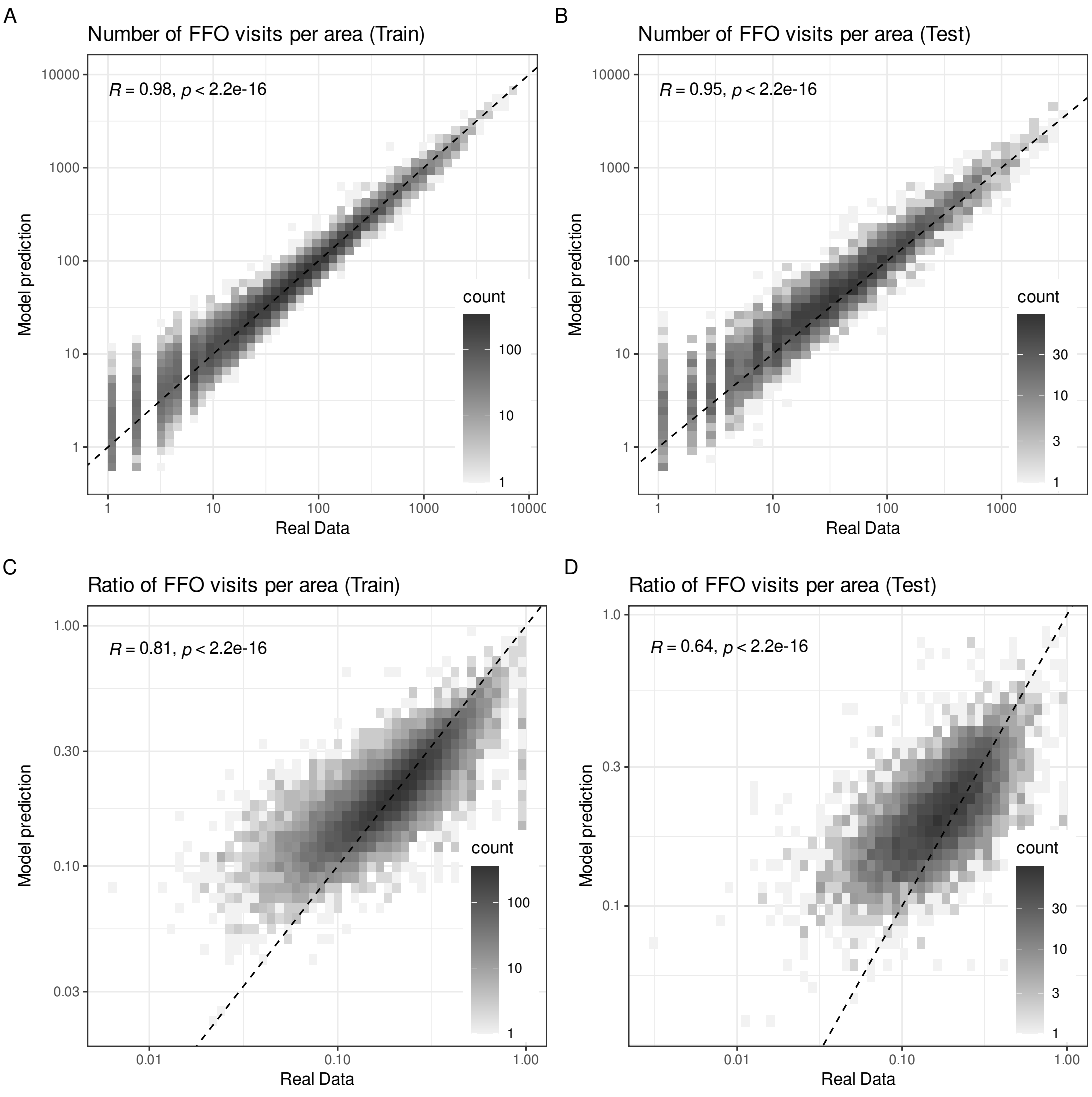

Figure S9: Correlation between the predictions for the total number of FFO visits (A and B) and fraction of FFO visits (C and D) of our model Eq. (2) and real data for the train (A and C) and test (B and D) sets in the two different out-of-sample strategies.

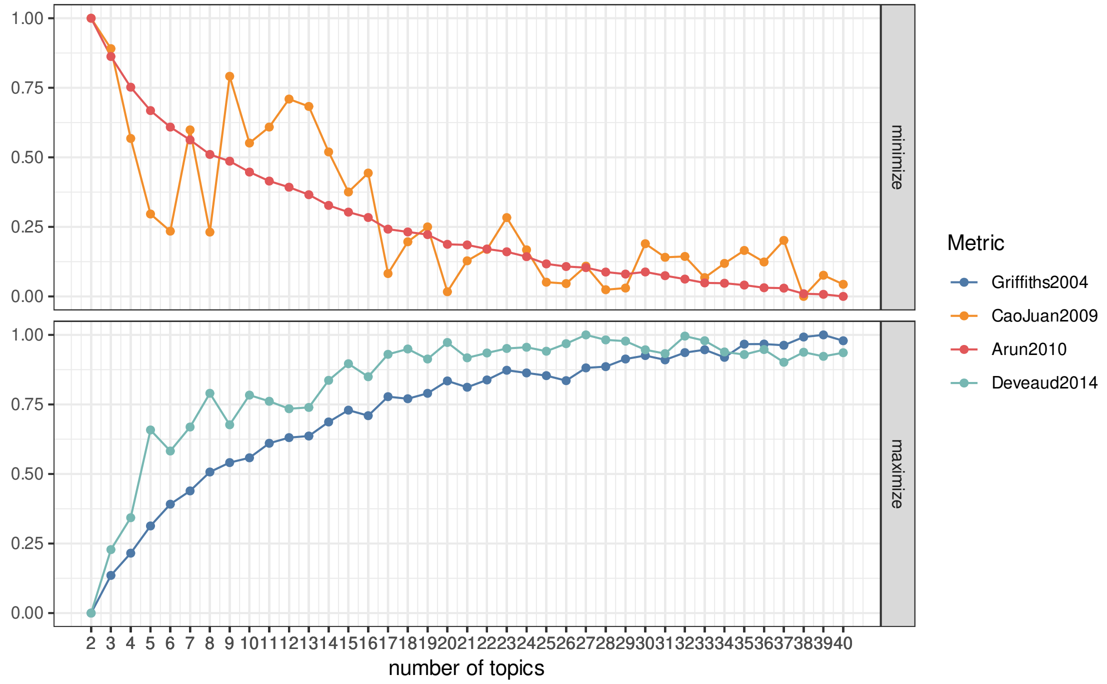

Figure S10: Variation of different metrics to estimate the optimal number of topics in the different census tracts. Metrics were obtained using the `ldatuning` package (23). As we can see, several metrics show a minimum/maximum around $k = 20$.

Table S7: Logistic regression results for the model in Equation (2) for all actions throughout the day and different out-of-sample test settings. The table presents the estimated coefficients with their respective standard errors in parentheses. p-values correspond to two-sided tests for the hypothesis that each coefficient is different from zero. We also report the Accuracy in train and test groups and its 95% confidence interval in square brackets.

Dependent Variable:	y_{it}	
Model:	Individual out-of-sample	Geographic out-of-sample
Variables		
$\phi(\mathbf{c}_{it})$	3.333	3.277
	(0.0252)	(0.0249)
	$p - value < 0.001$	$p - value < 0.001$
Fixed-effects		
day	Yes	Yes
user	Yes	Yes
Fit statistics		
Observations	8,598,339	8,513,588
Accuracy (Train)	0.8045	0.8064
	[0.8042,0.8048]	[0.8062,0.8067]
Accuracy (Test)	0.8016	0.7766
	[0.8011, 0.802]	[0.7761, 0.7771]

areas, education, and recreational areas, to more local ones related to different types of neighborhood groups of POIs.

Each census tract can be described then by the set of frequencies of each topic within them. Most of the census tracts cannot be described uniquely by a single topic but by a weighted composition of them. As expected, we find that topics like "University" or "Airport" or "Seaside" are less frequent in census tracts than others like "Garage", "Shopping Center" or "Neighborhood", see figure S12.

In summary, our LDA method allowed us to characterize each census tract by the patterns or groups of different POIs we could find there. It is interesting to see that the POIs in almost twenty thousand census tracts can be described by only 20 different groups of POIs that co-occur frequently in our urban areas. Most of those groups/topics are related to commercial activities, but some of them describe working places, recreational or health areas too.

Supplementary Note 9: Effect of interventions on other health risk groups

Here we investigate the possibility that our different interventions target only specific health risk groups. To this end, we use the PLACES Local data for Better Health dataset from the CDC (5) that contains model-based census tract-level estimates for different health risk behaviors and outcomes. Using those estimates, we classify each individual into different groups of health risk depending on which quantile their census tract is with respect to that risk. Figure S13 shows the total increase in the number of non-FFO visits for all interventions by health risk group: Obesity, Diabetes, Sleeping less than 7 hours, Levels of Physical Inactivity, and High Cholesterol. As we can see, the environmental-behavioral intervention always increases the number of non-FFO visits across different groups with

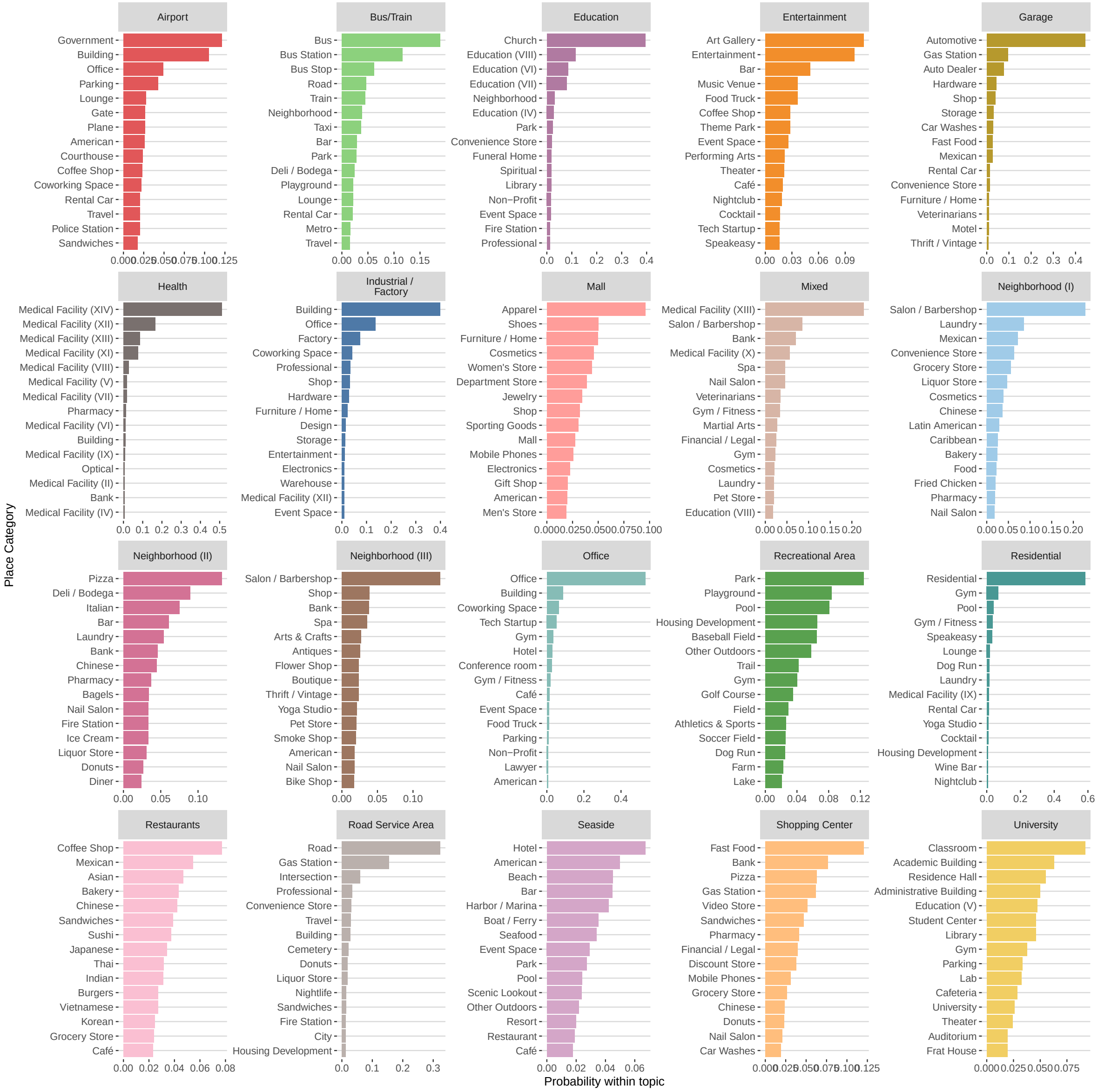

Figure S11: Composition of the different $k = 20$ POI topics found in our census tracts. We have given names to each of them for easy recognition. Bars represent the probability of finding each POI category in each of the topics. Note: no mobility data or visits were used in this analysis, only the spatial distribution of POIs from the Foursquare API.

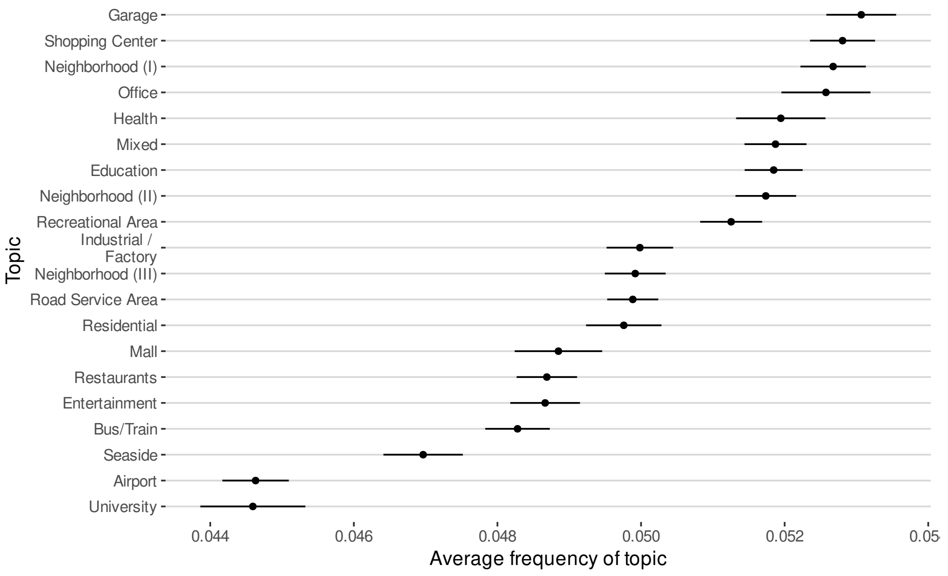

Figure S12: Average (and standard error of the mean) frequency of each topic across all census tracts in our dataset.

respect to the rest of the interventions. For example, compared with other interventions, we get 2x-2.5x more non-FFO visits on our environmental-behavioral intervention for groups coming from areas of high prevalence of diabetes and obesity. Note, however, that some other interventions have the same or even less effect on high-risk groups. For example, the "Food Swamp" and "Food Hotspots" interventions yield similar (low) effects in the high obesity, and high diabetes groups but very different in low obesity, and diabetes groups. These results show that, although some other interventions might have a heterogeneous impact across different health risk groups, our "Environmental-Behavioral" intervention always affects significantly those with high health risks, and significantly more than all other interventions.

Supplementary Note 10: Main results by city

In this section, we stratify the main results of our paper to investigate the differences and similarities in the effect of mobile food environments in FFO visits by metropolitan area. Figure S14 show the effect in the different cities is large and scattered around the value for the general model. Note, however, that the variability of β (log-odds) can be explained mostly by the fact that $\phi(\mathbf{c}_{it})$, the fraction of FFO around the contexts where decisions are made, is very different across cities. Since β measures the increase in the relative probability of going to FFO to not going to FFO for lunch when changing the fraction of FFO a given quantity, it is natural to expect that β is larger in cities where contexts have less FFO. In summary, although we see differences in the actual value of β by city, the differences in behavior by city appear to be largely explained by city-specific differences in the context of the mobile food contexts before lunch visits, rather than being due to intrinsic city-specific differences in people's food outlet visitation patterns.

On the other hand, we have also tested the effect of different interventions in each city. Results for the change of 20 FFO into non-FFO restaurants are presented in figure S15 where we can see again

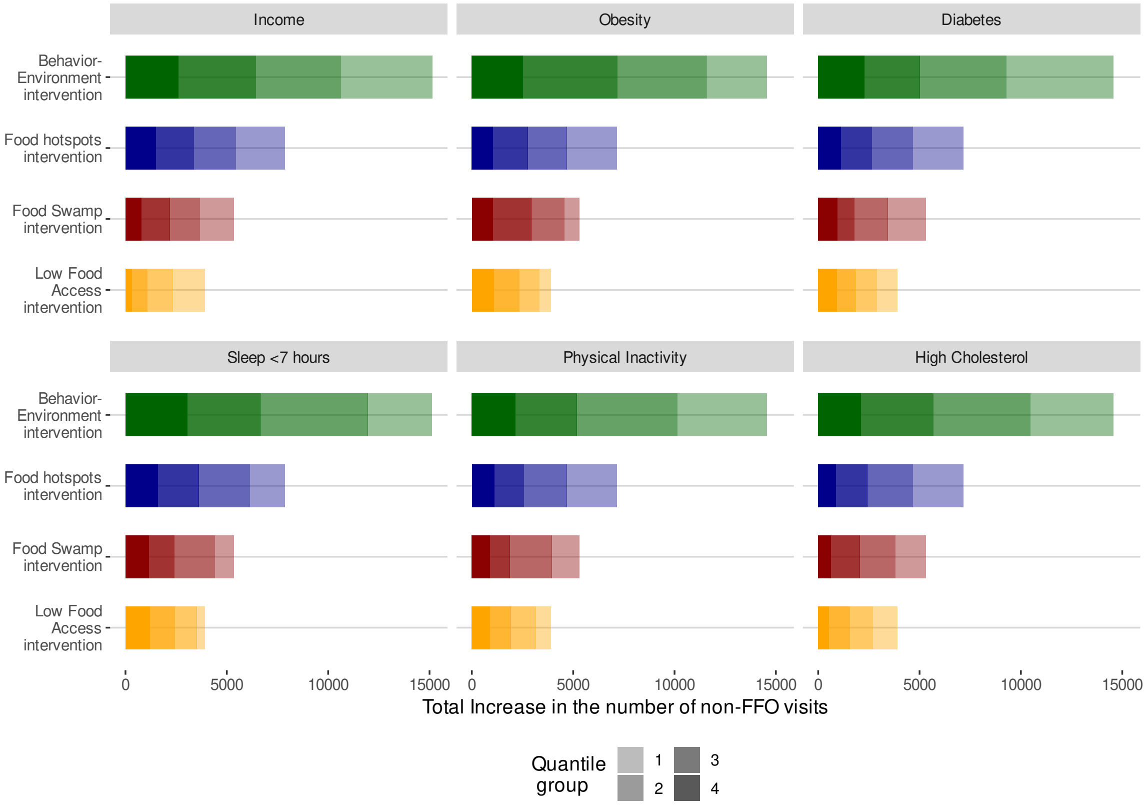

Figure S13: Effect of the intervention strategies on the different health outcomes groups. Each panel shows the total increase in the number of non-FFO visits after the intervention for users belonging to different health risk groups (shades). We classify each individual into different groups of health outcomes depending on which quantile their census tract is with respect to that outcome (darker shading for higher quantiles of each health outcome group). Health outcomes by census tract are obtained from the PLACES Local data for Better Health dataset from the CDC (5).

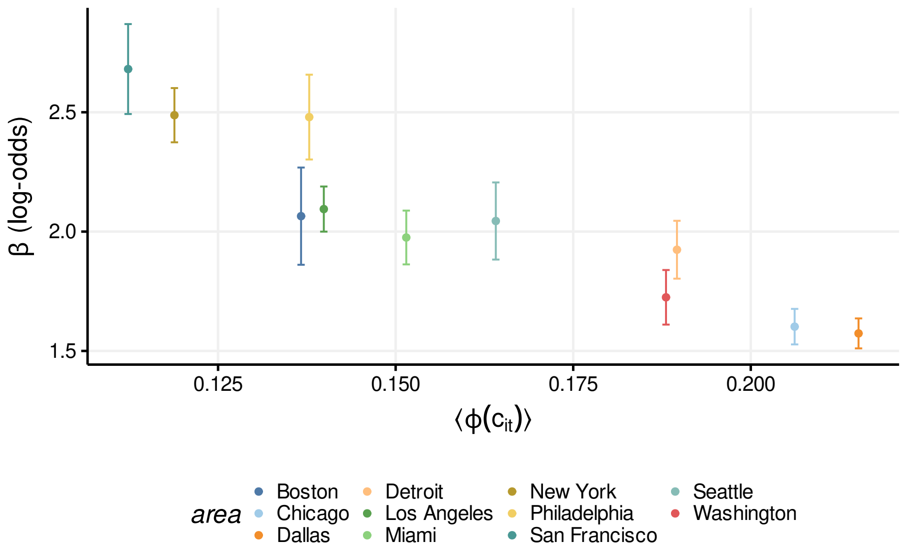

Figure S14: Effect of the mobile food context ϕ in choosing and FFO using the logistic regression Eq. (2) by city as a function of the average value of $\phi(\mathbf{c}_{it})$ by city. Error bars for the mean of ϕ by city are smaller than the symbol size.

that Behavior-Environment interventions are always more efficient than other interventions. Note, however, that the differences between strategies vary by city: for example, we find that Behavior-Environment interventions are particularly more effective than the rest in cities like Boston, Chicago, and Washington DC. In other cities like Philadelphia or Seattle, Behavior-Environment interventions are still more efficient than Food Hotspots but less than in the former group of cities. In all cases, though, Behavior-Environment interventions are much more efficient than the more traditional Food Swamp and Low Food Access interventions. Thus, our main result in the paper is also valid within particular cities.

Supplementary Note 11: Impact of mobile food environments for other types of food.

Given the strong effect of mobile food environments on visits to FFO, are our results similar for other types of food or do they only apply to fast food? To test this, we have calculated the effect of mobile food environments on visits to Asian and Latin American food outlets. Results for the model (2) in those cases are presented in Figure S16, where we can see that the results are very similar to visits to fast food outlets. We note that the log-odds coefficients are larger for Asian and Latin American food than for Fast Food. We identify Asian FO as those belonging to the categories Chinese, Thai, Malay, Asian, Dim Sum, Korean, Sushi, Vietnamese, Burmese, Noodles, Cambodian, Dumplings, Cantonese, Szechuan, Peking Duck, Ramen. Latin American outlets are those in the Mexican, Burritos, Arepas, Latin American, South American, Caribbean, Brazilian, Peruvian, Argentinian, Cuban, Colombian, Spanish, and Venezuelan categories.

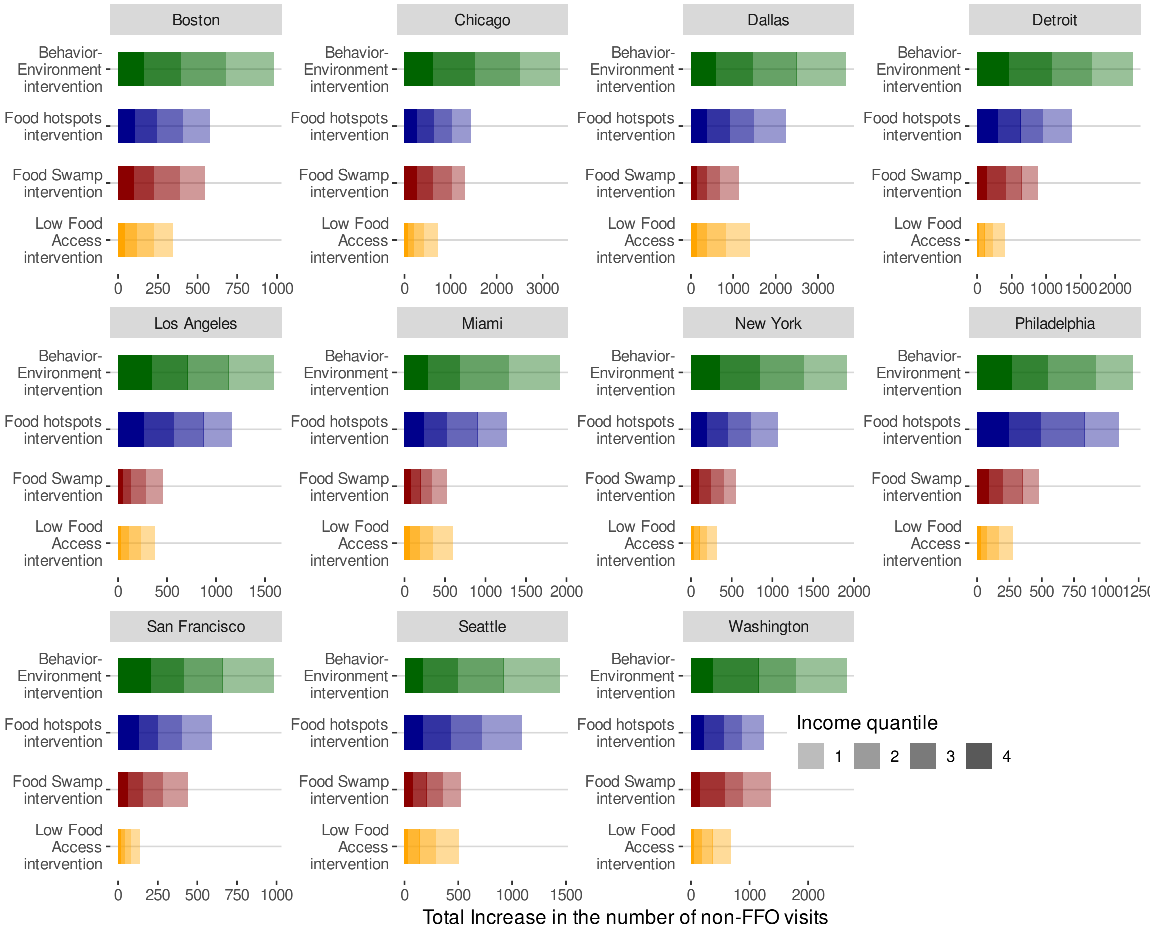

Figure S15: Total effect of each intervention strategy in each different city. Shades correspond to the number of actions changed by different income quantiles. See Figure 4 in the main text for more information.

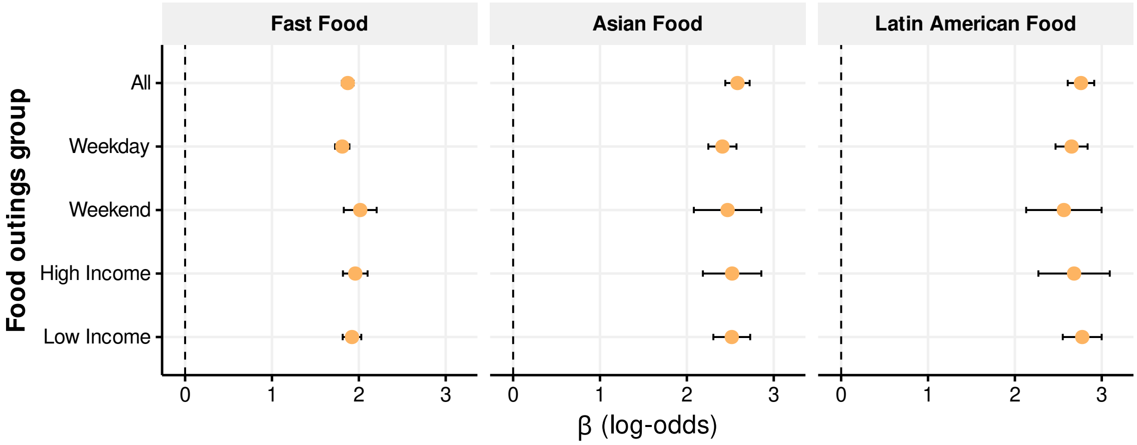

Figure S16: Effect of the mobile food context in choosing a food outlet at lunchtime for different types of food. Values show the coefficient β (log-odds) in Eq. (2), and bars indicate its standard deviation. The main result in the paper corresponds to the "Fast Food" type.

As in the previous section, this is due to the smaller values of the fraction of those food outlets around lunch contexts [$\langle \phi(\mathbf{c}_{it}\rangle$ = 0.089 for Asian and 0.081 for Latin American] than for fast food [$\langle \phi(\mathbf{c}_{it}\rangle$ = 0.162].

Supplementary Note 12: Robustness checks

12.1 Different definitions of POI visit

Visits to POIs are determined by looking at the closest POI to any stay up to a radius of $d^{max} = 200$ meters. Although we set $d^{max} = 200$m, we find that the median distance of stays attributed to POI is 27.40 m (Interquantil Range, IQR, 13.52 - 58.31), and the median distance of a stay attributed to FO is 19.15 m (IQR, 9.90 - 37.43m), and the median distance of a stay attributed to FFO is 17.77m (IQR, 9.40 - 31.82m). All of them are much smaller than $d^{max} = 200$m. In any case, we have tested the robustness of our results to this attribution of stays to POI by exploring other values of d^{max} from 20m to 200m. Again, the actual median distance to FO for those other values is much smaller than d^{max}: median of 10.18m (IQR, 6.19 - 14.58m) for $d^{max} = 20$m, 15.45m (IQR, 8.53 - 25.56m) for $d^{max} = 50$m, and 17.72m (IQR, 9.39 - 32.17m) for $d^{max} = 100$m. As we can see in Figure S17, we find very slight fluctuations with respect to d^{max} of our results on the effect of the context on Fast Food visits. These results indicate that our main findings are largely independent of and robust to the details of our approach for attributing stays to POI.

12.2 Different definitions of the context

We define the context $\mathbf{c}_{it}$ before lunchtime as the last stay before 11:30. We have also tested the sensitivity of our result to other definitions of the context before lunch. For example, we tested taking $\mathbf{c}_{it}$ as the longest stay before lunchtime, or the last one with a duration longer than 1 hour. The results

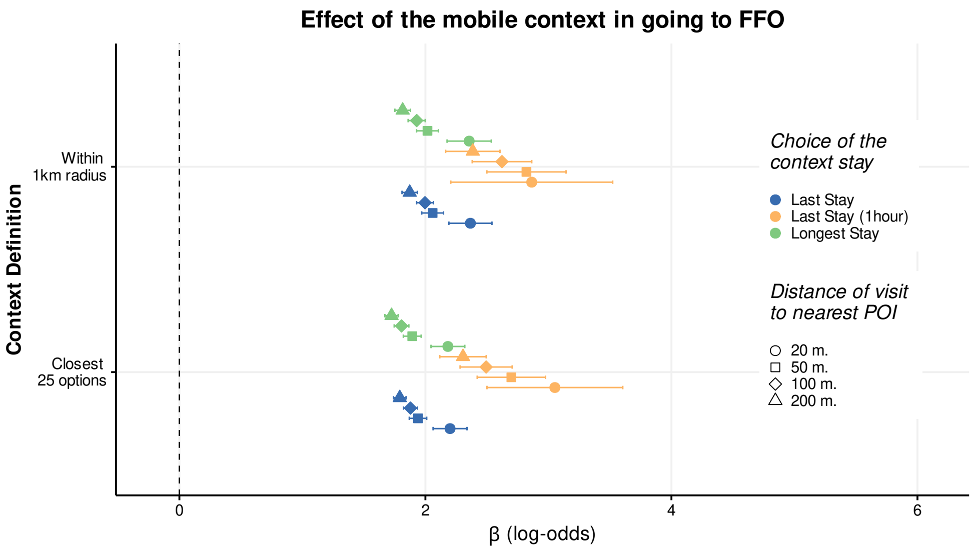

Figure S17: Effect of the mobile phone context in choosing a FFO when going to a food place at lunchtime for different definitions of FFO for different attributions of the stays to the POI and the choice of the stay for the context before lunch. Values show the coefficient β of the logistic regression (log-odds) in Eq. 2) and bars indicate its standard deviation. The result in the main paper corresponds to 200m, within a 1km radius and last stay.

for the logistic regression are presented in Figure S17 where we can see that different definitions of $\mathbf{c}_{it}$ yield very similar results on the effect of the context in fast food visits. These results indicate that our main findings are largely independent of and robust to the details of the definition of the context.

12.3 Different characterization of the context

To characterize the mobile food environment through the variable $\phi(\mathbf{x})$, we have taken the ratio of FFO to FO in a radius of 1km around place $\mathbf{x}$. However, in highly dense areas with many FO, a radius of 1km can include many FO, and it will be less likely that people are using the whole information about that large area when making their decision. Thus, we have tested the sensitivity of our results to other definitions of $\phi(\mathbf{x})$, which account for the different densities of FO in different areas. In particular, we have tested taking $\phi(\mathbf{x})$ as the ratio of FFO to FO of the closest 25 FO to $\mathbf{x}$, a definition that takes into account the density of FO around a place. As we can see in Figure S17, our results are very similar for both definitions.

We have also tested the possibility that rather than the absolute value of the fraction of FFO, users' decisions are conditioned by the relative number of FFO in the context to the whole city or county where the decision is made. That is that the users made decisions according to the excess of FFO in an area relative to the FFO in the whole county or city. To this end, we have modified model (2) as follows:

$$\Pr(y_{it} = 1) = \text{logit}^{-1}[\beta_0 + \alpha_i + \delta_t + \beta\phi(\mathbf{c}_{it}) + A_{it}] \quad (5)$$

where A_{it} is the county or the MSA where the decision is made. Results for the different areas are shown in table S8 where we can see that the results of model Eq. (5) are very similar to those presented

Table S8: Logistic regression results for the model in Equation (2) for all actions throughout the day and different out-of-sample test settings. The table presents the estimated coefficients with their respective standard errors in parentheses. p-values correspond to two-sided tests for the hypothesis that each coefficient is different from zero. We also report the Accuracy in train and test groups and its 95% confidence interval in square brackets.

Dependent Variable:		y_{it}	
Model:	A_{it} = County$_{it}$	A_{it} = MSA$_{it}$	No area
Variables			
$\phi(\mathbf{c}_{it})$	1.754	1.872	1.872
	(0.0339)	(0.0336)	(0.0335)
	$p < 0.001$	$p < 0.001$	$p < 0.001$
Fixed-effects			
Day	Yes	Yes	Yes
User	Yes	Yes	Yes
County	Yes		
MSA		Yes	
Fit statistics			
Observations	1,483,379	1,483,379	1,483,379
Squared Correlation	0.18894	0.18822	0.18820
Pseudo R^2	0.16123	0.16055	0.16054
BIC	4,438,643.7	4,438,410.0	4,438,285.7

Clustered (day & user) standard-errors in parentheses

in the main paper using model Eq. (2).

These results indicate that our main findings are largely independent of and robust to the details of the characterization of the context.

12.4 Different sets of FO and FFO

Since there is little consensus about what is the definition of FFO, we test the robustness of our results to different definitions of food and fast food venues. In particular, we use three different definitions:

- WF (Wikipedia and Foursquare) definition, the one used in the main paper and described in Section Supplementary Note 3:.
- MF (Manual and Foursquare) definition, the one used in (16) for the Los Angeles metropolitan area, see Section Supplementary Note 3:.
- WF+ (Wikipedia and Foursquare extended), which is similar to the WF definition but also adds in all independent restaurants that have the words “pizza” or “burger” in their names, as was done in (7).

As we can see in Figure S18, our results for the effect of the mobile food environment on the individual decision to visit FFO are very similar across the different definitions. We note, however, that the effect seems to be larger for the MF definition, due to the restrictive definition made in (16) for an outlet

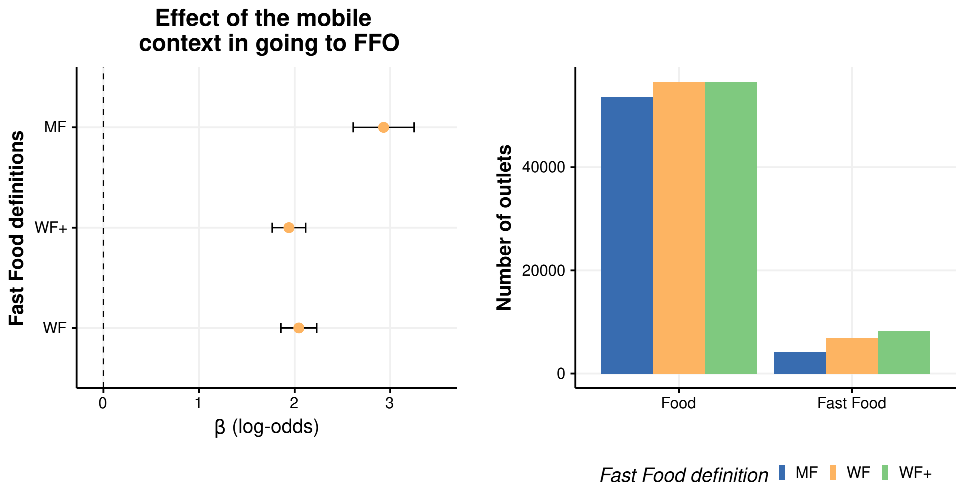

Figure S18: Left: Effect of the mobile phone context in choosing a FFO when going to a food place at lunchtime for different definitions of FFO. The analysis is only made for the Los Angeles metropolitan area since the manual definition in (16) is only available there. Values show the coefficient of the logistic regression (log-odds) in Eq. (2), and bars indicate its standard deviation. Right: Number of FO and FFO in the different definitions of fast food outlets.

to be considered as FFO. This makes the variable ϕ smaller in the different contexts and thus, the coefficient in the model gets larger.

Supplementary Note 13: Interventions

We want to identify those areas to intervene by changing how individuals evaluate the options around them. For example, this can be done by changing the number of FFO around them or by ranking differently the restaurants in the area on recommendation platforms. Here we want to identify those areas where those changes could have more impact. Our model predicts that the probability of choosing FFO is given by

$$P(y_{it}=1)=\frac{1}{1+e^{\beta_0-\delta_t-\alpha_i-\beta\phi(\mathbf{c}_{it})}}. \tag{6}$$

We want to investigate those places in which the change in that probability is larger after implementing an intervention I, that is, where the derivative with respect to I

$$\frac{\delta P(y_{it}=1)}{\delta \mathrm{I}}=\beta\frac{e^{X_{it}}}{(1+e^{X_{it}})^2}\frac{\delta\phi}{\delta \mathrm{I}} \tag{7}$$

is larger. Here $X_{it}=\beta_0+\delta_t+\alpha_i+\beta\phi(\mathbf{c}_{it})$. If we focus on a specific area Ω, then $\mathbf{c}_{it}$ is more or less the same within an area Ω, then we get that the change in that area is

$$\Delta^{\mathrm{FFO}}(\Omega)=\sum_{\mathbf{c}_{it}\in\Omega}\beta\frac{e^{X_{it}}}{(1+e^{X_{it}})^2}\frac{\delta\phi}{\delta \mathrm{I}} \tag{8}$$

This expression tells us that the change in probability of going to FFO in an area after changing the context around it depends on three things: i) the people that go to that area and their preferences (through α_i), ii) also on the number of actions there, and of course, iii) the context around that area. For example, we can have an area in which many people go, but they have a large individual preference for non-FFO ($\alpha_i \ll 0$), and they are not influenced by the FFO around. In that case, the impact of the intervention I will be small. Or an area in which not many people go, but they are very influenced by the FFO around ($\alpha_i \simeq 0$), so an intervention can change a large number of decisions.

Decreasing one unit of FFO in the area

Suppose we have an intervention where we decrease the number of FFO in area Ω by one unit (see the main test for possible interventions to accomplish this). In that case, $\phi(\mathbf{c}_{it})$ will change approximately by $-1/n_\Omega$, where n_Ω is the number of FO in an area Ω. Therefore we get that $\delta\phi/\delta\mathrm{I} \simeq -1/n_\Omega$ and the total reduction in the number of FFO visits of that intervention in the area Ω is

$$\Delta^{\mathrm{FFO}}(\Omega) = \sum_{\mathbf{c}_{it}\in\Omega} \frac{\delta P(y_{it}=1)}{\delta \mathrm{I}} = -\sum_{\mathbf{c}_{it}\in\Omega} \frac{\beta}{n_\Omega} \frac{e^{X_{it}}}{(1+e^{X_{it}})^2} \tag{9}$$

Supplementary Note 14: Libraries used

Analysis was conducted in R (25) using the following packages:

- Logistic and least square regressions with fixed effects were done using the `fixest` library (2).
- Random forest fits were performed using the `h2o` package (21).
- BSTS models were implemented using the `CausalImpact` package (4).
- Change of context was done using the `changepoint` (19).
- Packages `ggplot2` (32), and `tmap` (27) were used in the visualizations.
- Spatial analysis was done using the `sf` (24) package.
- LDA topic modeling was done using the `topicmodels` package (13).
- Approximate string matching for the classification of food outlets was done using the `stringdist` package (29).
- Access to the Census API was done using the `tidycensus`(31) package. Boundaries of the Census Block Groups were obtained from the Census API using the `tigris` (30) package.
- Regression tables were prepared using the `stargazer` package (15).

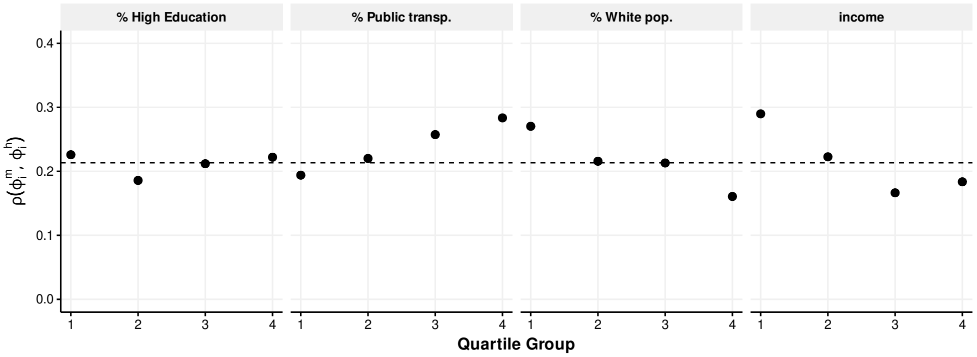

Figure S19: Correlation between home and mobile food environments for different demographic groups. Each group corresponds to a different quartile in each metropolitan area for the % of population with High Education, % of population using public transportation for work, % White population, and median household income. Quartile group 1 correspond to the lowest value and group 4 to the largest value of those demographic traits.

References

[1] United States Census Bureau. Core-Based Statistical Areas. `https://www.census.gov/topics/housing/housing-patterns/about/core-based-statistical-areas.html`, 2000. Accessed: 22-06-2019.

[2] Laurent Bergé. Efficient estimation of maximum likelihood models with multiple fixed-effects: the R package FENmlm. *CREA Discussion Papers*, (13), 2018.

[3] David M Blei, Andrew Y Ng, and Michael I Jordan. Latent dirichlet allocation. *Journal of machine Learning research*, 3(Jan):993–1022, 2003.

[4] Kay H. Brodersen, Fabian Gallusser, Jim Koehler, Nicolas Remy, and Steven L. Scott. Inferring causal impact using bayesian structural time-series models. *Annals of Applied Statistics*, 9:247–274, 2015.

[5] Centers for Disease Control and Prevention. 500 Cities: local data for better health. `https://www.cdc.gov/places/about/500-cities-2016-2019/index.html`, 2017. Accessed: 08-01-2021.

[6] William Cohen, Pradeep Ravikumar, and Stephen Fienberg. A comparison of string metrics for matching names and records. In *Kdd workshop on data cleaning and object consolidation*, volume 3, pages 73–78, 2003.

[7] Janet Currie, Stefano DellaVigna, Enrico Moretti, and Vikram Pathania. The Effect of Fast Food Restaurants on Obesity and Weight Gain. *American Economic Journal: Economic Policy*, 2(3):32–63, 2010.

[8] Andrea Cuttone, Sune Lehmann, and Jakob Eg Larsen. Inferring human mobility from sparse low accuracy mobile sensing data. In *Proceedings of the 2014 ACM International Joint Conference on Pervasive and Ubiquitous Computing: Adjunct Publication*, pages 995–1004, 2014.

[9] Ashlesha Datar, Amy Mahler, and Nancy Nicosia. Association of exposure to communities with high obesity with body type norms and obesity risk among teenagers. *JAMA Network Open*, 3(3):e200846–e200846, 2020.

[10] Economic Research Service (ERS), U.S. Department of Agriculture (USDA). Food environment atlas. `https://www.ers.usda.gov/data-products/food-environment-atlas/`, 2022. Accessed: 2022-02-04.

[11] Martin Ester, Hans-Peter Kriegel, Jörg Sander, Xiaowei Xu, et al. A density-based algorithm for discovering clusters in large spatial databases with noise. In *KDD*, volume 96, pages 226–231, 1996.

[12] Foursquare. Foursquare Venue Category Hierarchy. `https://developer.foursquare.com/docs/build-with-foursquare/categories/`, 2020. Accessed: 09-12-2020.

[13] Bettina Grün and Kurt Hornik. topicmodels: An R package for fitting topic models. *Journal of Statistical Software*, 40(13):1–30, 2011.

[14] Ramaswamy Hariharan and Kentaro Toyama. Project lachesis: parsing and modeling location histories. In *International Conference on Geographic Information Science*, pages 106–124. Springer, 2004.

[15] Marek Hlavac. *stargazer: Well-Formatted Regression and Summary Statistics Tables*. Central European Labour Studies Institute (CELSI), Bratislava, Slovakia, 2018. R package version 5.2.2.

[16] Abigail L. Horn, Brooke M. Bell, Bernardo Garcia Bulle Bueno, Mohsen Bahrami, Burcin Bozkaya, Yan Cui, John P. Wilson, Alex Pentland, Esteban Moro, and Kayla de la Haye. Investigating mobility-based fast food outlet visits as indicators of dietary intake and diet-related disease. *medRxiv*, 2021.

[17] Infogroup. Infogroup US Historical Business Data, 2016.

[18] Rebecca Killick and Idris A. Eckley. changepoint: An R Package for Changepoint Analysis. *Journal of Statistical Software*, 58(3):1–19, 2014.

[19] Rebecca Killick, Kaylea Haynes, and Idris A. Eckley. *changepoint: An R package for changepoint analysis*, 2022. R package version 2.2.3.

[20] Kaitlin Kirasich, Trace Smith, and Bivin Sadler. Random forest vs logistic regression: binary classification for heterogeneous datasets. *SMU Data Science Review*, 1(3):9, 2018.

[21] Erin LeDell, Navdeep Gill, Spencer Aiello, Anqi Fu, Arno Candel, Cliff Click, Tom Kraljevic, Tomas Nykodym, Patrick Aboyoun, Michal Kurka, and Michal Malohlava. *h2o: R Interface for the 'H2O' Scalable Machine Learning Platform*, 2022. R package version 3.38.0.1.

[22] Esteban Moro, Dan Calacci, Xiaowen Dong, and Alex Pentland. Mobility patterns are associated with experienced income segregation in large US cities. *Nature Communications*, 12(1):4633, 2021.

[23] Murzintcev Nikita. *ldatuning: Tuning of the Latent Dirichlet Allocation Models Parameters*, 2020. R package version 1.0.2.

[24] Edzer Pebesma. Simple Features for R: Standardized Support for Spatial Vector Data. *The R Journal*, 10(1):439–446, 2018.

[25] R Core Team. *R: A Language and Environment for Statistical Computing*. R Foundation for Statistical Computing, Vienna, Austria, 2020.

[26] Matthew Salganik. *Bit by bit: Social research in the digital age*. Princeton University Press, 2019.

[27] Martijn Tennekes. tmap: Thematic maps in R. *Journal of Statistical Software*, 84(6):1–39, 2018.

[28] United States Census Bureau. 2013-2017 American Community Survey 5-year Estimates. `https://www.census.gov/programs-surveys/acs`, 2019. Accessed: 2020-12-04.

[29] M.P.J. van der Loo. The stringdist package for approximate string matching. *The R Journal*, 6:111–122, 2014.

[30] Kyle Walker. *tigris: Load Census TIGER/Line Shapefiles*, 2020. R package version 1.0.

[31] Kyle Walker and Matt Herman. *tidycensus: Load US Census Boundary and Attribute Data as 'tidyverse' and 'sf'-Ready Data Frames*, 2020. R package version 0.10.2.

[32] Hadley Wickham. *ggplot2: Elegant Graphics for Data Analysis*. Springer-Verlag New York, 2016. R package version 3.3.2.

[33] Wikipedia. List of fast food restaurant chains in the US. `https://en.wikipedia.org/wiki/List_of_fast_food_restaurant_chains#United_States`, 2023. Accessed: 2023-08-11.
