# Supplementary material for: Effect of mobile food environments on fast food visits
Source: Nat Commun. 2024 Mar 14;15:2291. doi: 10.1038/s41467-024-46425-2 (PMC10937966; doi:10.1038/s41467-024-46425-2)
Supplement: Supplementary file 3 — Reporting Summary [file 41467_2024_46425_MOESM3_ESM.pdf]

nature portfolio

Corresponding author(s): Esteban Moro

Last updated by author(s): Jan 30, 2024

Reporting Summary

Nature Portfolio wishes to improve the reproducibility of the work that we publish. This form provides structure for consistency and transparency in reporting. For further information on Nature Portfolio policies, see our Editorial Policies and the Editorial Policy Checklist.

Statistics

For all statistical analyses, confirm that the following items are present in the figure legend, table legend, main text, or Methods section.

n/a	Confirmed	
☐	☒	The exact sample size (*n*) for each experimental group/condition, given as a discrete number and unit of measurement
☐	☒	A statement on whether measurements were taken from distinct samples or whether the same sample was measured repeatedly
☐	☒	The statistical test(s) used AND whether they are one- or two-sided<br>*Only common tests should be described solely by name; describe more complex techniques in the Methods section.*
☐	☒	A description of all covariates tested
☐	☒	A description of any assumptions or corrections, such as tests of normality and adjustment for multiple comparisons
☐	☒	A full description of the statistical parameters including central tendency (e.g. means) or other basic estimates (e.g. regression coefficient) AND variation (e.g. standard deviation) or associated estimates of uncertainty (e.g. confidence intervals)
☐	☒	For null hypothesis testing, the test statistic (e.g. *F*, *t*, *r*) with confidence intervals, effect sizes, degrees of freedom and *P* value noted<br>*Give P values as exact values whenever suitable.*
☐	☒	For Bayesian analysis, information on the choice of priors and Markov chain Monte Carlo settings
☐	☒	For hierarchical and complex designs, identification of the appropriate level for tests and full reporting of outcomes
☐	☒	Estimates of effect sizes (e.g. Cohen's *d*, Pearson's *r*), indicating how they were calculated

Our web collection on statistics for biologists contains articles on many of the points above.

Software and code

Policy information about availability of computer code

Data collection	No special software was used to collect the data
Data analysis	Data analysis was done using R and different packages. See Supp. Material for a description of them. Code to run the analysis has been deposited on GitHub https://github.com/emoro/mobile_food_environments

For manuscripts utilizing custom algorithms or software that are central to the research but not yet described in published literature, software must be made available to editors and reviewers. We strongly encourage code deposition in a community repository (e.g. GitHub). See the Nature Portfolio guidelines for submitting code & software for further information.

Data

Policy information about availability of data

All manuscripts must include a data availability statement. This statement should provide the following information, where applicable:

- Accession codes, unique identifiers, or web links for publicly available datasets
- A description of any restrictions on data availability
- For clinical datasets or third party data, please ensure that the statement adheres to our policy

The data that support the findings of this study are available from Spectus through their Social Impact program https://spectus.ai/social-impact/, but restrictions apply to the availability of these data, which were used under the license for the current study and are therefore not publicly available. Anonymized data to reproduce the results of our paper is available on request from https://doi.org/10.5281/zenodo.7798632. Other data used come from the American Community

Survey (5y) from the Census https://www.census.gov/ programs-surveys/acs, the PLACES Local data for Better Health from the CDC https://www.cdc.gov/places/about/ 500-cities-2016-2019/index.html), or the Food Environment Atlas from the U.S. Deparment of Agriculture (https://www.ers.usda.gov/data-products/food-environment-atlas) which are publicly available on their websites. A description of these datasets is given in Supplementary Note 1.

Research involving human participants, their data, or biological material

Policy information about studies with human participants or human data. See also policy information about sex, gender (identity/presentation), and sexual orientation and race, ethnicity and racism.

Reporting on sex and gender	Since we cannot access that information about our users for privacy reasons, we do not have any findings related to gender or sex for individuals.
Reporting on race, ethnicity, or other socially relevant groupings	Since we cannot access that information about our users for privacy reasons, we do not have any findings related to race or other social relevant groupings for individuals.
Population characteristics	See below
Recruitment	See below
Ethics oversight	The privacy-enhanced mobility data was collected by the company Spectus using anonymized records of GPS locations from users that opted-in to share the data anonymously through a General Data Protection Regulation (GDPR) and California Consumer Privacy Act (CCPA) compliant framework. Additionally, we obtained IRB exemption to use the mobility data from the MIT IRB office. (COUHES protocol #1812635935 and its extension #E-2962)

Note that full information on the approval of the study protocol must also be provided in the manuscript.

Field-specific reporting

Please select the one below that is the best fit for your research. If you are not sure, read the appropriate sections before making your selection.

☐ Life sciences ☒ Behavioural & social sciences ☐ Ecological, evolutionary & environmental sciences

For a reference copy of the document with all sections, see nature.com/documents/nr-reporting-summary-flat.pdf

Behavioural & social sciences study design

All studies must disclose on these points even when the disclosure is negative.

Study description	We perfomed a quantitative study on observational data using statistical methods to detect behaviors around fast food consumption. Logistic regression and semi-causal (Bayesian) techniques were used to detect the influence of food environments to visit fast food places
Research sample	Data used are geo-locations from anonymous opted-in devices collected by the company Spectus in 11 metro areas in the US. Data has been aggregated at the level of places, categories or census areas where a number of devices are present to prevent de-anonymization. There is not sampling, the dataset was provided by the Company and it includes all the dataset.
Sampling strategy	There is no sampling of the data. We use all the data provided by the company. To check that the sample size was sufficient, we used post-stratification techniques has been used to correct for potential biases in the sample of users and to ensure population representation (see Supp. Materials)
Data collection	Data collection was done by the company Spectus
Timing	Data was collected from October 2016 to March 2017
Data exclusions	No data was excluded
Non-participation	Only anonymous opted-in devices where used in the analysis
Randomization	As we said, the data was provided by Spectus and it is not an experiment. Thus we don't have to apply randomization techniques.

Reporting for specific materials, systems and methods

We require information from authors about some types of materials, experimental systems and methods used in many studies. Here, indicate whether each material, system or method listed is relevant to your study. If you are not sure if a list item applies to your research, read the appropriate section before selecting a response.

Materials & experimental systems

n/a	Involved in the study
☒	☐ Antibodies
☒	☐ Eukaryotic cell lines
☒	☐ Palaeontology and archaeology
☒	☐ Animals and other organisms
☒	☐ Clinical data
☒	☐ Dual use research of concern
☒	☐ Plants

Methods

n/a	Involved in the study
☒	☐ ChIP-seq
☒	☐ Flow cytometry
☒	☐ MRI-based neuroimaging
